# Molecules, Mechanisms, and Disorders of Self-Domestication: Keys for Understanding Emotional and Social Communication from an Evolutionary Perspective

**DOI:** 10.3390/biom11010002

**Published:** 2020-12-22

**Authors:** Goran Šimić, Vana Vukić, Janja Kopić, Željka Krsnik, Patrick R. Hof

**Affiliations:** 1 Department of Neuroscience, Croatian Institute for Brain Research, University of Zagreb Medical School, 10000 Zagreb, Croatia; vukic.vana@gmail.com (V.V.); janjakopi5@gmail.com (J.K.); zkrsnik@hiim.hr (Ž.K.); 2 Nash Family Department of Neuroscience, Friedman Brain Institute, and Ronald M. Loeb Center for Alzheimer’s disease, Icahn School of Medicine at Mount Sinai, New York, NY 10029, USA; patrick.hof@mssm.edu

**Keywords:** chemoattractants, chemorepellents, epithelial-mesenchymal transition (EMT), extracellular matrix molecules, fibroblast growth factor (FGF), methyl-CpG-binding protein 2 (MeCP2), neural crest cells (NCCs), self-domestication, thyroid hormones, vascular endothelial growth factor (VEGF)

## Abstract

The neural crest hypothesis states that the phenotypic features of the domestication syndrome are due to a reduced number or disruption of neural crest cells (NCCs) migration, as these cells differentiate at their final destinations and proliferate into different tissues whose activity is reduced by domestication. Comparing the phenotypic characteristics of modern and prehistoric man, it is clear that during their recent evolutionary past, humans also went through a process of self-domestication with a simultaneous prolongation of the period of socialization. This has led to the development of social abilities and skills, especially language, as well as neoteny. Disorders of neural crest cell development and migration lead to many different conditions such as Waardenburg syndrome, Hirschsprung disease, fetal alcohol syndrome, DiGeorge and Treacher-Collins syndrome, for which the mechanisms are already relatively well-known. However, for others, such as Williams-Beuren syndrome and schizophrenia that have the characteristics of hyperdomestication, and autism spectrum disorders, and 7dupASD syndrome that have the characteristics of hypodomestication, much less is known. Thus, deciphering the biological determinants of disordered self-domestication has great potential for elucidating the normal and disturbed ontogenesis of humans, as well as for the understanding of evolution of mammals in general.

## 1. The Concept of Self-Domestication and Its Possible Dependance on Neural Crest Cells (NCCs)

Good cooperation requires efficient social and emotional communication. The linguistic communication in humans is unmatched by any other animal species. Compared to apes, even non-linguistic human communication skills are superior to all other hominin. Moreover, children who have not yet adopted language and speech skills will outperform adult apes in cooperative communication. For example, although we know that chimpanzees often use gestures in several different ways, there is no single documented case of a chimpanzee pointing at another animal or object with a finger. Children can do this by the end of the first year of life. Therefore, we can conclude that over the last 5–7 million years, a significant divergence in social and emotional intelligence between humans and other hominins (chimpanzees [*Pan troglodytes*, Blumenbach, 1775] and bonobos [*Pan paniscus*, Schwartz, 1929]) has taken place. According to the self-domestication hypothesis, the main change that occurred during this evolutionary period had an emotional character: a human became more tolerant of another human, as well as significantly less aggressive and frightened in his presence. Observed from this perspective, human evolution is, in part, similar to that of the dog and other domesticated species. Consequently, appreciating the process of domestication is important for understanding emotional and social communication in humans.

The term domestication implies the process through which different organisms adapt to life in the human environment. The terms domesticated plants, animals and other organisms refer to all living beings whose original (wild) phenotype has been modified by cultivation and life under the control of humans through multiple generations. Humans have thus domesticated a few dozen species for various reasons: most often, these species were “cultivated” as a source of food (e.g., agricultural crops) or valuable raw materials (e.g., wool from sheep), some were domesticated for work assistance (e.g., horse or cattle), and some simply to be pets or ornamental plants.

Charles Darwin was first to describe the domestication phenomenon in his book “*The Variation of Plants and Animals Under Domestication*”, published in 1868 [1]. Darwin observed that all domesticated mammals share a set of common morphological, physiological, and behavioral traits that cannot be seen in their wild ancestors. Today, the domestication syndrome in mammals encompasses about fifty different phenotypic traits that a domesticated species has in relation to its wild ancestors. The ten most noticeable are: (1) weaker pigmentation (especially white “spots” in fur); (2) smaller ears; (3) lopped (drooped) ears due to weaker ear cartilage; (4) smaller muzzle; (5) smaller teeth; (6) weaker expression of the emotion of fear and fear-associated reactive aggression (= tameness, docility), along with underactive sympathetic system and delayed maturation of the adrenal gland with reduced production of stress hormones (adrenaline, noradrenaline and cortisol) and a weaker “fight or flight response”; (7) smaller brain size (together with reduced skull volume as the skull volume depends on size of the brain); (8) shortened reproductive cycle (more frequent estrus) and longer gestation period; (9) delayed maturation of the hypothalamic-pituitary-adrenal axis, which prolongs the period of socialization and slows down the process of sexual maturation, while retaining certain juvenile characteristics in adult organism (the process called neoteny, sometimes also pedomorphosis or fetalization); and (10) smaller and curly tail due to weaker tail cartilage [2,3].

Of the ten main phenotypic features of the domestication syndrome listed above, one characteristic is always present in all domesticated species: the feature no. 6—tameness, the reduced aggression towards humans as a result of reduced acute fear and chronic stress due to the presence of humans. Other phenotypic characteristics, depending on the species, may or may not be present (or they may be present to a greater or lesser extent). Darwin described seven of these ten phenotypic traits of domesticated species (those under numbers 1, 2, 4-6, 8, and 10). However, it should be noted that today’s dogs have numerous morphological changes caused by the intensive creation of individual breeds in the last 150–200 years. These changes do not correlate with behavioral changes and are not associated with domestication [4]. Therefore, they should not be confused with the initial domestication of the gray wolf (*Canis lupus*) by nomadic hunters at least 15,000 years ago, when wolf was selected for hunting based solely on reduced aggression (it is believed that other features emerged as an accompanying phenomenon). Paleogenomic findings suggest that such a selection occurred approximately 15,000 years ago, while initial selection most likely began as early as 20–40,000 years ago [5,6].

The observation that a set of different phenotypic traits consistently emerges and is subsequently inherited in different species of domesticated mammals, even in birds and fish, posed a great challenge to Darwin that he wanted to explain. As he published his observations on domestication only two years after Gregor Mendel’s published work on plant hybridization [7], which is considered as the beginning of genetics, the biological basis of the domestication phenomenon is still considered the oldest, still unsolved problem in genetics. Mendel’s work was printed in a total of 115 copies, and since he greatly appreciated Darwin, Mendel sent one of his 40 copies to Darwin. Unfortunately, although Darwin himself crossed edible peas too and came up with a ratio close to 3:1 in the F2 generation (90:37 in favor of the dominant versus recessive form), it seems that Darwin never read Mendel’s work [8]. Therefore, until his death in 1882, he thought that offspring always contain mixed parental traits, implying that traits could not be quantified in offspring [8].

Darwin proposed two possible explanations for the phenomenon of domestication [1]. His first hypothesis was based on the observation that during domestication, the captivated animals ate higher-quality food (provided by humans) and generally lived in better conditions, which could thereafter somehow cause phenotypic changes. This assumption was pure speculation, as Darwin could not find any evidence on the extent to which this environmental impact was important in each subsequent generation, or the extent to which it influenced the inheritance of acquired domestication traits. Nevertheless, the prediction arose from this hypothesis, noting that domesticated animals would lose the traits obtained by domestication upon returning to their wild habitats. Although it was possible to verify this assumption at the time, definitive evidence was difficult to obtain due to the reduced survival rate of domesticated animals once released into the wild, as well as because they cross-reproduced in the wild with their wild relatives. However, contemporary research has refuted the validity of such an assumption. One of the most convincing evidence was the finding that reduced brain and skull sizes in domesticated rodents, dogs, and other species whose domesticated individuals were returned to the wild (the process of feralization, generally a much less studied process opposite to domestication) remained the same, e.g., in the case of the American otter (*Mustela vison*) after as long as 40 generations of life in the wild [9]. Moreover, a fairly large number of domestication features can be seen in those mammalian species that have evolved in isolation on the islands [3].

Darwin’s second assumption by which he tried to explain the domestication phenomenon was that phenotypic traits in domesticated animals arose as the result of their mixing with other breeds. This hypothesis is interesting as it is true that mixing different breeds can create new phenotypic traits. Still, it does not explain why exactly the same “set” of phenotypic traits of domestication always emerge in so many different species. This second Darwin’s assumption has also been refuted over time. Namely, the domestication process was carried out experimentally with individual wild animals that could not mix with other breeds, and yet despite this limitation, it led to a complete domestication syndrome. The largest study of this kind was initiated in 1959 by Dimitry K. Belyaev and Lyudmila N. Trut in Novosibirsk, Russia, when they began to domesticate the silver fox (melanistic form of the red fox, *Vulpes vulpes*) [10]. Only 10% of the calmest cubs were selected in each subsequent generation of foxes that were allowed to mate. The only screening criterion was tameness. The cubs were then raised as pets, and their phenotypic traits were monitored. After four years, i.e., after the fourth generation of domesticated silver foxes (they mate once a year), initial changes in the phenotype could be observed, which indicated commencement of the domestication syndrome. After the ninth generation, at the age of three months, some cubs had lopped (drooped) ears and a curly tail, and their muzzles were getting shorter and rounder (so their appearance was becoming more juvenile; muzzle changes are today believed to occur mostly due to changes in the structure and regulation of the *bmp3* gene [osteogenin]) [11]. Nowadays, these foxes are as obedient as most dog breeds, and even look more like dogs than foxes.

Since 1970, besides screening for tameness, researchers in Novosibirsk started to perform screening of aggressive animals to compare them with domesticated animals in regard to gene expression in different areas of the nervous system and other characteristics [12,13]. With this long-term experiment, Belyaev confirmed that selection for tameness is the only sufficient prerequisite for achieving a complete domestication syndrome. After the 15th generation, the measured cortisol levels in the blood were approximately 50% lower in domesticated foxes than in those that lived in the wild, and their adrenal glands were decreased in size [11]. Consequently, Belyaev believed that, under protected environmental conditions created by humans, animals exhibit have lower levels of stress that subsequently promote the change in the pattern of gene expression (so-called “destabilizing selection” theory) through a weaker secretion of stress hormones (adrenaline, noradrenaline, and especially cortisol) [14]. In other words, endocrine changes influence early development, thus affecting subsequent (later) behavior, meaning that some initially epigenetic changes become permanent over time. Transgenerational epigenetic inheritance occurs largely as a result of the influence of various toxins and dietary regimes (such as prenatal famine) [15,16,17,18], and although it has been poorly studied in domesticated (domestic) animals, it still cannot explain the domestication syndrome.

Trut and her collaborators have partly followed the reasoning of Belyaev in an attempt to explain these data. It is based on the hypothesis that the domestication syndrome may occur due to genetic (mutations, polymorphisms) and epigenetic changes that occur in early development (e.g., under the influence of hormones) before activation of genes involved in the putative specific networks whose altered expression leads to phenotypic traits of domestication [19,20,21]. However, the recent research has largely discarded the hypotheses of Belyaev (weaker secretion of stress hormones) and Trut (fewer genes that are essential in early development whose action changes under differential hormonal influence) as probable explanations for the occurrence of domestication syndrome. Specifically, following a similar procedure as described in foxes, experimental domestication was repeated in some other animal species, such as weasel and rat, and the phenotypic characteristics of the domestication syndrome did not occur in sequence or independently of each other under the influence of hormones. Also, it is highly unlikely that a very small network of the same genes and their mutations or epigenetic changes could directly control all of the diverse, simultaneously occurring characteristics of the domestication syndrome. However, even if those assumptions were true, one would expect those temporary or permanent genetic changes in a smaller number of genes during the earliest periods of development to lead to much more serious consequences or to affect concomitantly a much greater proportion of cells throughout the body. Therefore, despite these valuable efforts, the mystery of domestication remains a matter of debate.

Although various other assumptions have been made in an attempt to find a unique explanation, such as that changes in the secretion of thyroid hormones or thyroid gland changes may be in the root of the domestication syndrome [22], which is an extension of the endocrinologic hypothesis of Belyaev, most researchers now agree that the neural crest hypothesis proposed by Wilkins and collaborators in 2014 is the closest to the solution [2]. When it comes to domestication of plants, which have neither a central nervous system nor a neural crest, the answer probably lies in related clusters of genes essential for the specification of quantitative traits [23].

The neural crest hypothesis is based on a reduced number, impaired migration, or poorer proliferative activity of cells originating from the neural crest as direct causes of the domestication syndrome. NCCs are a transient group of embryonic cells that arise from the dorsal part of the neural tube during early embryonic development, and are unique to vertebrates (they do not exist in invertebrates) [24,25]. As they enabled the development of cranial sensory placodes with neurogenic potential from which sensory and parasympathetic ganglia of the head developed, as well as numerous other neurogenic (e.g., parts of the olfactory epithelium and the inner ear) [26] and non-neurogenic (e.g., lens, auditory ossicles, adenohypophysis) derivatives, it can be concluded that NCCs provided vertebrates with a predatory lifestyle [21,24]. In other words, neural crest-derived cells enabled the collection of key information from the skin such as touch, pain, and temperature, as well as later specialization of sensory organs (sense of smell, taste, sharper vision, hearing, and balance) [27].

Although all of these cells are direct derivatives of the ectoderm, due to their multipotency, long migration pathways, and capacity to differentiate into a large number of different cell types, they are often referred to as the fourth germ layer, in addition to the ectoderm, mesoderm, and endoderm [28,29]. It is important to emphasize that for the proper development and migration of NCCs, many other surrounding cells, as well as distant cells, including adjacent neural plate and mesoderm cells, but also ectodermal cells that are not associated with the development of the central nervous system, are of vital importance. These different cell types and their activity are important because of the many reciprocal interactions of which we will single out two.

**Figure 1:**
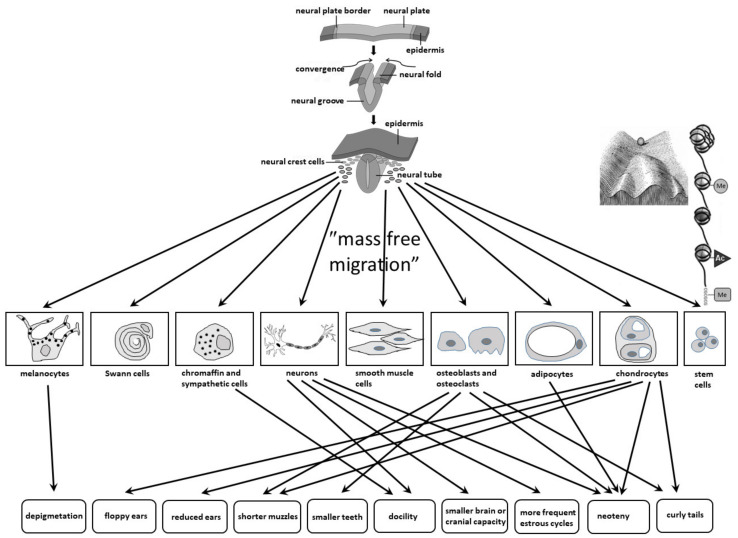
Schematic representation of neural tube development, neural crest cells (NCCs) migration, and some associations of NCCs with phenotypic traits of the domestication syndrome in mammals. The upper part of the schematics is made according to Marieb and Hoehn (2018) [27] and Kaltschmidt et al. (2018) [28]. The Waddington’s epigenetic landscape (“*canalization*”) on the right side illustrates contribution of epigenetic changes on NCCs migration, differentiation, and proliferation, and is made according to Strobl-Mazzulla and Bronner (2014) [29]. For the purpose of this schematic representation, no distinction is made between anterior (cephalic) and posterior somatic NCCs.

Firstly, although the cells of the neural crest are not directly involved in the construction of the central nervous system, it is known that they induce the development of the parts of the limbic and frontal lobes by secreting certain growth factors (see later). Secondly, many other cells, those in the immediate vicinity but also the ones in the remote areas, secrete signaling molecules that serve as chemoattractants and chemorepellents to properly guide NCCs to their final destinations. This goal is usually very distant, and additionally, unlike, for example, the migration of neuroblasts from the ventricular and subventricular zones whose destination is the cerebral cortex, in this type of migration, there are no radial glial cells that would serve as a ladder to “climb” to their final destinations (target tissues and organs). Therefore, the migration of NCCs is also called “mass free migration”. The overly simplified schematics of development and migration of NCCs are shown in Figure 1.

Wilkins et al. stated three possible reasons for the neural crest cell deficits that are responsible for domestication syndrome: (1) initially reduced numbers of NCCs formed, (2) fewer migratory capabilities of NCCs with their consequently lower numbers at the final (target destination) sites, and (3) reduced ability to proliferate once they reach their destinations, and proposed that the migration defects are probably particularly important [2].

## 2. Basic Biology of NCCs

**Figure 2:**
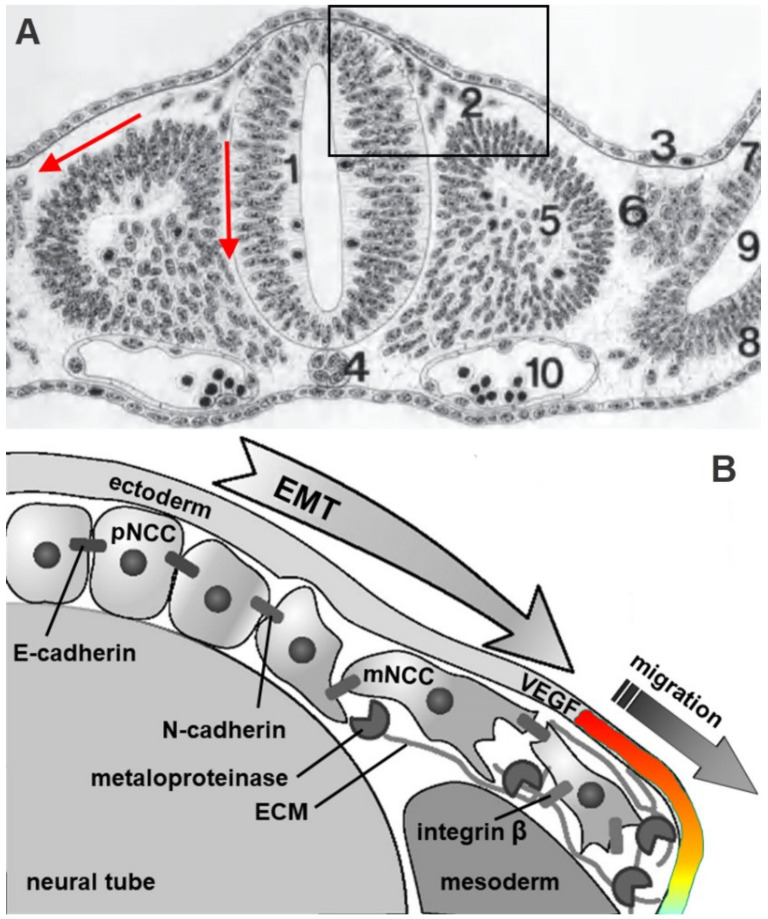
Delamination of NCCs from neuroectoderm via ectodermal-mesenchymal transition and migration of NCCs via ventrodorsal and ventromedial pathways. Cross-section through a human embryo on day 24 of gestation (crown-rump length 3 mm). The lower part of figure (**B**) represents enlarged inset from the upper part (**A**). Legend: 1, neural tube; 2, neural crest migratory cells; 3, ectoderm; 4, dorsal chord (notochord); 5, somite; 6, intermediate mesoderm; 7, somatopleura; 8, splanchnopleura; 9, intraembryonic whole; 10, aorta; a red medial (vertical) arrow between the neural tube and the somite indicates the ventromedial direction of NCCs migration, a red lateral arrow between the ectoderm and the somite indicates the dorsolateral direction of NCCs migration; ECM, extracellular matrix; EMT, epithelial-mesenchymal transition; mNCC, migratory neural crest cells; pNCC, premigratory neural crest cells, VEGF, vascular endothelial growth factor. The spatio-temporal expression pattern of VEGF in the ectoderm (colored) regulates the NCCs migratory front. The upper part of figure (**A**) is from Christ (1985) [32], and the lower part of the image is schematized according to Szabo and Mayor (2018) [31] and McLennan et al. (2010) [33].

The first description of the neural crest in a chicken embryo was made by Wilhelm His in 1868 [30]. During human embryonic development, the neural crest first appears in the rostral portion of the neural tube on the 19th day of gestation. After initial induction, the neural crest is physically separated from the neuroectoderm by a delamination process [31]. Delamination is mediated by a complex process of ectodermal-mesenchymal transition that allows NCCs cells to become less adhesive and mobile (Figure 2).

Anteroposterior positional identity of premigratory NCCs in all vertebrates is established by the nested and combinatorial expression of the homeodomain transcription factors of the *HOX* (homeobox) gene family, the same molecular mechanism that controls segmentation and patterning of the rhombomeres from which progenitor NCCs delaminate. Thus, a distinction should be made between the anterior (or cephalic) HOX-negative NCCs, more posterior cephalic NCCs, which maintain the segment-specific HOX codes of their original rhombomeres, and posterior somatic NCCs. As cephalic NCCs give rise to most cranial bones, they are essential for the shape of the skull and face and therefore likely critical for the phenomenon of self-domestication (see below).

**Figure 3:**
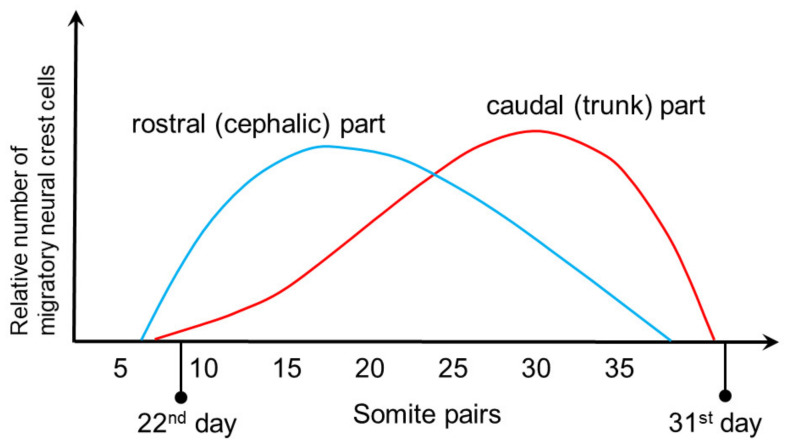
Schematic representation of the dynamics of neural crest cell migration in the rostral and caudal part of the human embryo. Migration begins around day 22 and ends by day 31 of gestation. The total number of somites in human is 37 as out of the original 42–44, some of the most caudal ones rapidly disappear [36].

After delamination, the migration of NCCs begins. Depending on their initial position, these cells migrate in two different morphological patterns: (1) in the area of the future head and neck, there is a cephalic “sheet-like” mass migration that begins on days 22/23 and reaches its peak somewhere between days 23 and 26 of gestation, and (2) in the trunk area there is migration in the form of traveling “caravans” of migrating neurons (“chain migration”), where total number of migrating cells reaches its peak between days 26 and 30 of gestation (Figure 3). Contacts between NCCs that migrate together are particularly pronounced in early migrating cells in the trunk area, as well as during the whole cephalic mass migration, where contacts established by thin filopodia at distances greater than the cell diameter allow them to have a “safer” journey, i.e., to reach their final destinations together [34].

While it is still largely unknown how NCCs migrate through multiple microenvironments and into specific targets, it has been shown in chick embryo that the invasion of cranial NCCs, specifically the rhombomere 4 migratory stream into branchial arch 2, occur due to chemoattraction through neuropilin-1 and vascular endothelial growth factor (VEGF) interactions, where the spatiotemporal expression pattern of VEGF in the ectoderm (Figure 2B) correlates with, and is therefore believed to regulate, the NCCs migratory front [35].

Far apart from what can be seen during the development of the cerebral cortex, where the newborn migrating neuroblasts use radial glial cells as scaffolds and travel along the radial glial fibers to reach their final destinations [37], the migration of NCCs is completely dependent on chemotaxis, i.e., the interaction of receptors expressed on them (Robo, ephrin, VEGF, endothelin and neuropilin receptors, CXC family of chemokine receptors, DAN/NBL1 receptors for bone morphogenetic proteins, and many others) with extracellular matrix molecules, especially collagen, laminin, fibronectin, endothelin, semaphorin, F-spondin, versican, and others [34,38].

Derivatives of multipotent NCCs include several lineages: (1) ectomesenchymal lineage, which gives rise to chondroblasts (chondrocytes), osteoblasts (osteocytes), fibroblasts, odontoblasts, mesenchymal cells of the heart, myoblasts, pericytes, smooth muscle cells of branchiogenic and brain blood vessels, tendons of all ocular and chewing muscles, connective tissue of all head and neck glands, meninges, stem cells of the bone marrow, and adipocytes, (2) nerve cell lineage, which gives rise to neurons of all sensory, spinal, parasympathetic, and sympathetic ganglia, satellite glial cells of all spinal, autonomic and sensory ganglia, Schwann and all other glial cells within the peripheral nervous system, spleen and pancreas, (3) lineage of secretory cells: glandular cells of the pineal gland, lacrimal and sublingual glands, enterochromaffin cells and chromaffin cells of the adrenal gland, thymus and thyroid stromal cells, parafollicular thyroid cells (which produce calcitonin), glomus cells and carotid body cells, and (4) lineage of pigment cells—melanocytes (including those in the iris).

NCCs migrating through the rostromedial half of the somite (medial vertical arrow in Figure 2A) differentiate into sensory and sympathetic neurons (ganglia) of the peripheral nervous system, aortopulmonary septum, atrioventricular valves, smooth muscles of the aortic arch, enteric autonomic nervous system, chromaffin cells of the adrenal glands, while cells that migrate dorsolaterally (lateral red arrow in Figure 2A) differentiate into pigment cells of the skin (melanocytes) [31,33].

Once they reach their final destination, further differentiation and final specification of neural crest migratory cells take place. This process also depends on morphogenic inducers, such as bone morphogenetic proteins (BMP), fibroblast growth factor (FGF), WNT (wingless + int-1), and Notch proteins, and others [35,39]. Only a small proportion (about 1–3%) of human NCCs have full stem cell pluripotency (e.g., they express NANOG and POU5F1, and the transcription factor SOX2), while most of them are multipotent for a short period of time or only unipotent progenitor cells [36].

Although it is still unclear which of these reasons for the reduced number of neural crest-derived cells would be the most important; their initial formation, impaired specification, migration or reduced proliferative activity (degree of division) once they reach their destinations (or a combination of all these three), it is well established that the tameness (docility) is the most important component for the domestication syndrome. Tameness is achieved through the overall reduction in the number of nerve cells of the sympathetic ganglia and adrenal medulla, as such change weakens the fight-or-flight response to a new or threatening stimulus [2].

As NCCs are not progenitors for any part of the central nervous system, the most difficult phenotypic feature of the domestication syndrome to explain was reduced brain size. For example, domestic pigs have a smaller brain volume by a third compared to a wild boar of the same body size, and brain size is similarly smaller in other domesticated mammals as opposed to their wild ancestors (rat, guinea pig, gerbil, rabbit, sheep, goat, cattle, yak, llama, camel, horse, donkey, ferret, cat, dog, mink, fox, and many other species) [9]. However, the results of the experiment Le Douarin et al. published in 2004 showed that NCCs play an indirect but crucial role in inducing the development of the frontal and limbic regions via FGF8 secretion. Namely, the lack of facial development and the reduction of the frontal areas caused by surgical removal of the neural crest in the early chick embryonic development could be fully compensated by the external addition of FGF8 protein [37].

Furthermore, it still seems difficult to explain why the reproductive (estrous/menstrual) cycle is shortened (in species with estrous cycles, females are generally sexually active only during the estrus phase whereas females of species with menstrual cycles can be sexually active at any time in their cycle, even when they are not about to ovulate). One possible explanation is a decrease in the activity of the hypothalamic-pituitary-gonadal axis. This assumption is based on the fact that this axis controls the reproductive cycle in all mammals, and its reduced activity leads to the shortening of the cycle. Although not considered a direct derivative of NCCs, another possibility that has not yet been proved is possible involvement (hypofunction) of the pineal gland in this process. However, it is known that the pineal gland plays a key role in the regulation of estrous in relation to the duration of daylight, and its reduced activity in domesticated foxes has been described [38].

**Figure 4:**
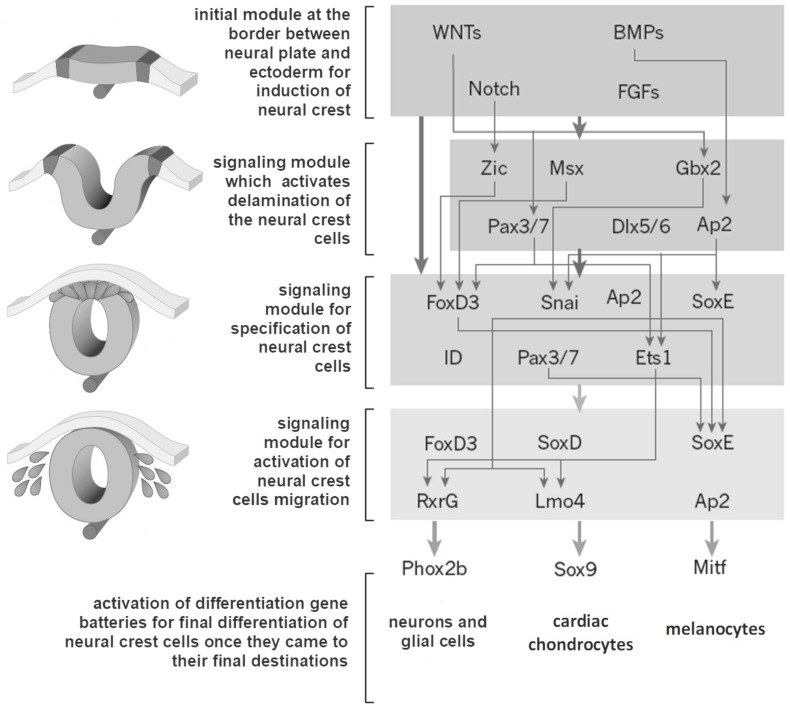
Simplified schematics of stepwise activation of the regulatory gene modules and networks for NCCs development, delamination, specification, migration, proliferation, and differentiation. Genes for specification of the first signaling module, which induces neural crest, do not exist in invertebrates. Scheme is modified according to Green et al. (2015) [39] and Simões-Costa et al. (2015) [35]. Today, it is known that the specification and migration modules include more genes, including *Twist*, *Sox5*, *Sox9*, *Myc*, *Tfap2*, *Sox10*, *Myb*, *RxrG*, *Myc*, and many others.

To sum up, it can be said that the neural crest hypothesis directly links embryonic developmental processes of neural crest cell migration and phenotypic traits of domestication, but still does not explain which genes, epigenetic processes, intercellular interactions, and signaling pathways are involved in domestication. It is therefore not surprising that the number of studies on genes playing important roles in the specification, migration, and postmigratory interactions of NCCs has increased significantly in recent years. In general, activations of groups of genes by particular transcription factors are considered to act as modules that determine the main steps that take place in each subsequent stage of neural crest cell development (Figure 4) [35,39].

## 3. Human NCC-Dependent Disorders

According to a recent review article, at least 66 human diseases whose underlying cause is an NCC disorder are relatively well-known to date [40]. Some of the most commonly mentioned are: Waardenburg syndrome—a condition that manifests with areas of skin, hair, and eyes without melanocytes and congenital sensorineural deafness [41,42,43], Hirschsprung’s disease or aganglionosis, where there are parts of the intestine without settled NCCs that differentiate into neurons of the enteric plexuses—Meissner’s submucosal and Auerbach’s myenteric (as they mediate peristaltic movements, the affected parts of the intestine where food accumulates must be surgically removed), fetal alcohol syndrome—alcohol consumed by the mother interferes with normal migration of NCCs, so the head and face circumference are reduced, the eyes are wide apart (hypertelorism), and there are other facial abnormalities of the nose, mouth, and ears, DiGeorge’s syndrome—congenital malformations of the face and heart associated with neurological and learning difficulties, including the predisposition to schizophrenia and bipolar disorder, Treacher-Collins syndrome—due to mutation of *TCOF1* gene malformations of the face, jaw, ears and eyes are seen, Kallmann’s syndrome—due to mutations of *FGF8* gene and gene for FGF receptor 1 (*FGFR1*) there is a lack of development of the terminal nerve whose fibers facilitate the migration of the olfactory stream neurons to the hypothalamus; as these neurons secrete gonadotropin-releasing hormone (GnRH), there is a reduced secretion of follicle-stimulating hormone (FSH) and luteinizing hormone (LH) from the adenohypophysis, resulting in hypogonadotropic hypogonadism, cleft palate, sensorineural hearing loss as well as partial or complete anosmia [40].

**Figure 5:**
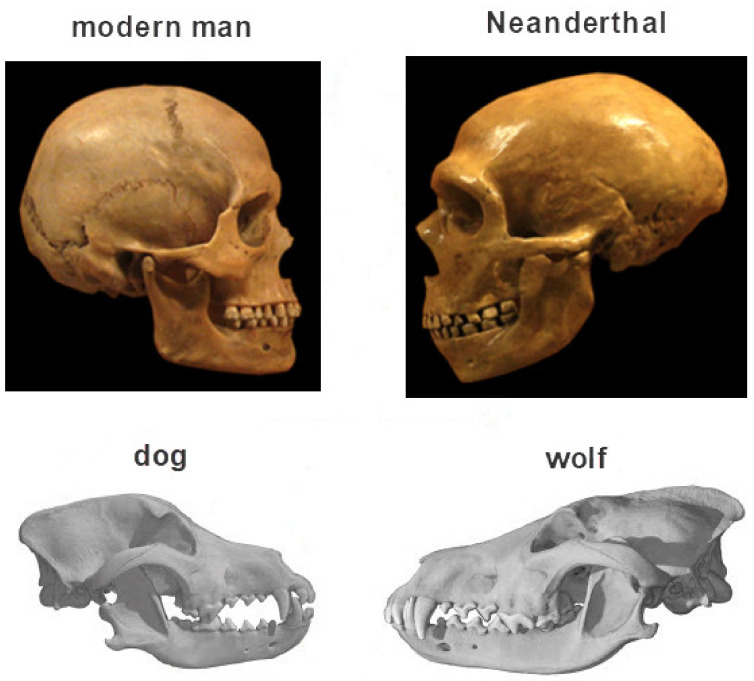
Comparison of dog domestication and human self-domestication. Illustration modified from Theofanopoulou et al. (2017) [45]. Photographs of the skull of modern man (*Homo sapiens*) and prehistoric man (*Homo neandertalensis*) are from commons.wikimedia.org. Similar to differences between dog and wolf, note that the skull of modern man is much more neotenic compared to the skull of a Neanderthal (smaller skull and brain, oval forehead, less protruded nasal bone, smaller teeth, orthognathia), which can be said to be gerontomorphic.

The idea that man is a domesticated primate was first elaborated by Darwin as early as 1871 in his book “*The descent of man, and selection in relation to sex*” [44]. Taking the fact that modern man has so many phenotypic features of domestication, especially those of the skull, face and teeth, as well as decreased aggression and prolonged retention of youthfulness (neoteny) along with comparison of the recent evolutionary past of human and dog, it is impossible not to notice that the process of self-domestication has also taken place in humans (Figure 5).

## 4. Neoteny

Neoteny (juvenilization, paedomorphosis) is a type of heterochrony and one of the main features of the domestication syndrome [46]. Neoteny is defined as a delay or slowing of physical development in relation to sexual development. The term neoteny usually refers to ontogenetic development, and pedomorphosis to the whole species. The word neoteny was coined by Julius Kollmann in 1853 [47], when he described the maturation of the Mexican salamander axolotl (“Mexican walking fish”, *Amb[l]ystoma mexicanum* and *Amb[l]ystoma tigrinum*) [46], which, unlike many other amphibians, for example a frog, remains in the tadpole stage with the possibility of swimming and reproduction throughout life (so-called complete neoteny) [47,48]. The axolotl has features of both fish (outer gills) and salamanders (four legs). If water is permanently available, it can live longer than 32 years. However, in the environment without water, axolotl would be irreversibly transformed into an adult form without gills and with larger legs, and its lifespan would be about five years [49]. Metamorphosis into an adult axolotl can also be experimentally induced by thyroid hormone or thyrotropin (thyroid-stimulating hormone, TSH) administration or by electrical stimulation of hypothalamic neurons [50,51]. An increase in the concentration of thyroid hormones [52], or increased expression of deiodinase, which converts free thyroxine (FT4) into the active form triiodothyronine (FT3) [51], determines the metamorphosis from tadpoles to adults not only in axolotls but also in frogs (such as in *Xenopus laevis* from the family *Pipidae*, which are one of the most common experimental models in science in general) and many other amphibians.

Today, it is known that all axolotl tissues express functional receptors for thyroid hormones, so the “failure” of transformation into an adult is most likely due to a very low level of thyroid hormone production due to poor secretion of thyrotropin-releasing hormone (TRH) from the hypothalamus and TSH release from the pituitary gland [50,51]. Hence the hypothesis that thyroid hormones may also be responsible for the domestication syndrome in mammals [22]. Obligatory neoteny is also seen in the olm (*Proteus anguinus*), which, in addition to its weight (only 15–20 g), resembles axolotls in many aspects (e.g., it has external gills and small legs). The olm is well adapted to long-term starvation [53], reaches sexual maturity at about 15.6 years of age, and lays about 35 eggs every 12.5 years. The average recorded life expectancy in captivity is 68.5 years, while the maximum life expectancy is estimated at 103 years [54]. Although its ontogenetic development stops very early and irreversibly, it is, unlike in the axolotl, not related to thyroid hormones [55]. Blind salamanders similar to the olm have also been described in North America (mudpuppy, *Necturus maculosus*), and live for about 30 years [56].

Many other species may experience partial neoteny that disappears after full maturation, e.g., in the goby species *Leucopsarion petersii* and *Gymnogobius urotaenia*, termites of the species *Kalotermes flavicollis*, dragonflies of the order *Ephemeroptera*, males of long-tailed manakin of the family Pipridae (species *Chiroxiphia linearis* and *Chiroxiphia caudata*), insects of the genus *Strepsiptera*, crustaceans of the *Ischnomesidae* family living in the depths of the ocean (species *Stylomesus hexapodus* and *Haplomesus corniculatus*), jellyfish *Turritopsis nutricula* (this species appears to be capable of multiple transformations (transdifferentiation) from mature stage to immature stage of polyp), aphids from the family *Aphididae* and many other species. Another outstanding example of neoteny is the naked mole rat (*Heterocephalus glaber*), which weighs only about 35 g and lives about thirty years or more. This is about 10 times longer compared to other species of rats, which have a maximum lifespan age about 3 years [49]. The naked rat has a high degree of resistance to hypoxia and oxidative stress [57], and so far, it has been described that it possesses at least 43 different neoteny features [49]. Of other mammals, neoteny is expressed in whales and humans, as well as in burdock of the genus *Sonchus* and plants of the genus *Echium* [49].

**Figure 6:**
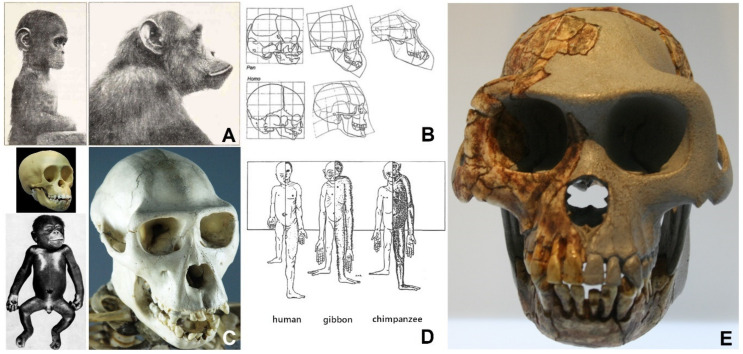
The development of the idea of neoteny in human. (**A**). Photograph of a chimpanzee child and an adult chimpanzee from Naef (1926) [60]. (**B**). The top row shows the growth and shape of the skull in a fetus, cub, and adult chimpanzee, and the bottom row shows the skull of a fetus and an adult human. The direction of transformation is the same: negative skull allometry is seen as the skull grow slower than the rest of the body and is therefore being proportionally smaller in adults than in children (hypoallometry) and positive allometry of the face and jaw growing faster than the rest of the body and therefore proportionally higher in adults than in children (hyperallometry); image from Starck and Kummer (1962) [61]. (**C**). Skulls of young and adult chimpanzees (from commons.wikimedia.org) for comparison with Figures A and B. In the lower left corner is a picture of a seven-month-old chimpanzee fetus. Hair growth can be seen on the head, in the eyebrow area, the edges of the eyelids, lips, and cheeks, i.e., in those places where we see them today in adults, which is considered as another proof of neoteny in human; image from the University of Wisconsin Public Digital Repository website (www.omnia.ie). (**D**). Comparative view of infant and adult human, gibbon, and chimpanzee, all aligned with height in sitting position (frame represents trunk and head size). Lesser apes (gibbons) have extremely long front limbs, even at birth. The legs always grow faster than the trunk, especially in a human. The newborn has relatively short arms at birth. Note smaller hands and feet in man. The image transferred from Verhulst (1993) [62], originally drawn by Schultz (1926) [63]. (**E**). Cast of a reconstructed skull of the *Ardipithecus ramidus*. Note the flattened face and pronounced orthognathia (the cast is on display at the State Archaeological Museum in Chemnitz, Federal Republic of Germany; image from commons.wikimedia.org).

According to Ashley Montagu, Kollmann used the term neoteny to express that the axolotl “retains the qualities of youth” [58]. Although it was not his original intention, the word neoteny corresponds very well to the fact that youthful traits are prolonged because, along with developed sexual maturity, they persist into adulthood [58]. Kollmann also suggested in 1905 that humans may have evolved from bonobo chimpanzees through a process of juvenilization, or neoteny, while chimps may have evolved through the juvenilization of apes [46,59]. This assumption was probably best illustrated by Adolf Naef in 1926 (Figure 6) [46,60].

Although Edward Drinker Cope was the first to propose it [46], the concept of juvenilization was largely developed by Lodewijk Bolk, who called it a “fetalization theory” [64,65,66,67]. In his work from 1927 [66] Bolk states the following list of features of human neoteny: (1) flattened and wide face and orthognathia (aligned upper and lower jaw), (2) reduction or absence of body and face hair, (3) pigment loss in skin, eyes and hair, (4) ear shape, (5) epicanthus (skin fold of the inner corner of the eye), (6) central position of large occipital opening (that moves backwards during ontogenetic development of primates), (7) relatively larger brain size (relative to body size), (8) retention of sutures between flat bones of the skull and spherical skull shape throughout life, (9) large labia in women, (10) hand and foot shape, (11) pelvic shape, (12) ventrally directed position of the vagina in women, (13) variations of teeth and skull sutures. Bolk added a number of additional features to the basic list: (14) absence/reduction of the supraorbital protrusion of the frontal bone, (15) absence of cranial ridges, (16) thinner skull bones, (17) change in the position of orbits within the cranial cavity (and larger eyes), (18) brachiocephaly, (19) smaller teeth (both in the upper and lower jaw), (20) later eruption of teeth, (21) lack of toe rotation, (22) prolonged period of dependence of the child on parents, (23) prolonged period of development, (24) prolonged (extended) lifetime, and (25) shorter limbs in relation to body size (with longer legs in relation to arms). Some of the listed characteristics are visible in Figure 6B,D.

Some features of neoteny such as orthognathia [68] can already be seen in the oldest known hominids discovered in 1994 in the Aramis area of Ethiopia, attributed to *Ardipithecus ramidus* (Figure 6E) [69]. Seventeen fossils of these australopithecines date from the period of 4.4 million years ago [70]. The morphology of the pelvic and femur bones of a female skeleton indicates that the Ardipithecus could climb trees but also walk upright like a human [71]. This was confirmed by an analysis of the morphology of the base of her skull, which determined its lateral extension with carotid openings shifted aside and with pronounced sphenoid angle, while the large occipital foramen laid more anteriorly [72]. Another study, which analyzed the morphological features of the skull of 41 humans (18 adults and 23 juveniles) and 50 chimpanzees (17 adults and 33 younger ages) using a three-dimensional generalized Procrustes analysis, confirmed neoteny of human skull [73]. Moreover, it has been established that neoteny of the skull in humans not only includes pedomorphosis of the shape, but also that neotenic changes in the relative growth rate of the skull are more pronounced in those parts that are in contact with the brain (in comparison with other parts of the skull that are not in direct contact with the brain) [73]. According to this analysis, these additional structural changes are related to upright gait (flexion of the cranial base, displacement of the large occipital opening closer to the palate), breathing (conspicuous nose), and chewing (reduced prognathism).

Although there is still not enough data and information to draw firm conclusions, many authors think that self-domestication has played a key role in human evolution as the driving force of self-domestication was probably the selection of lower levels of aggression for greater social tolerance, i.e., community life. Within the whole concept of self-domestication, many prominent evolutionary biologists consider neoteny as the most important feature [46,58,74]. Since not all the facts are available, as stated earlier, there are numerous theories and interpretations. One of them states that during the recent human evolutionary past neoteny was closely related to sexual selection and became more important over time as an increasing proportion of women began to live after menopause. As these women could no longer give birth this may have led to a greater degree of selection for phenotypic traits of youth, associated to female fertility, by men. Although such theories have a number of shortcomings and limitations, especially those arising from cultural differences, recent research confirms that men are more attracted to women who have neotenic features: smaller skeleton, narrower joints, reduced hair, lower basal metabolism, higher-pitched voice, larger tear ducts, larger eyes, small nose, full lips, among others [58,75]. Similar studies have shown that among women who work as models, there are more of those with phenotypic characteristics of neoteny [75]. Similarly, neotenic features in men also have advantages, probably in making them look less threatening and thus more socially acceptable, which may increase their attractiveness [76].

The second explication posits the neoteny as crucial to achieving a greater capacity for emotional communication, for which facial expressions are the most important. In this sense, facial hair loss has led to more efficient and better communication of socially important messages based on emotional signaling by facial expressions [77]. A third and likely far-reaching interpretation states that the neotenic brain has extended the time-window of plasticity of neural networks in humans. In other words, his extended childhood allowed him a longer period of learning and acquiring new skills, curious exploration of the world around him, and better shaping of all cognitive processes, especially social, linguistic, and emotional [46,74,78]. This is especially true for the neoteny of synaptic spines of the association pyramidal neurons of the frontal cerebral cortex [79]. Namely, excessive generation and developmental remodeling of synaptic spines continues after adolescence, in the second half of the second and in the third decade of life before complete stabilization in adult values. This has given humans unprecedented opportunities to reach the highest levels of cognitive abilities, while burdening them with increased susceptibility for the development of abnormal neural circuits in adolescence and post-adolescence that are manifested by neuropsychiatric disorders such as schizophrenia (see below) [79].

This finding is further supported by the following evidence: (1) as many as 48% of genes associated with the development of the human dorsolateral prefrontal cortex are significantly differently expressed or have a significantly different expression curve in humans relative to the frontal lobe of chimpanzee and rhesus monkey (*Macaca mulatta*) [80], (2) when comparing the expression profiles of the cerebral cortex genes in human, chimpanzee and rhesus monkey, of the 169 genes showing significantly different expression, approximately 90% are more pronounced in humans (as opposed to the heart or liver where the numbers of more or less pronounced genes are approximately equal) [81]. Other studies comparing the brains of humans, chimpanzees, and other hominids also show that changes in human brain evolution were not limited to a simple increase in size (total area of the prefrontal cortex in humans is 181.4 cm^2^ and chimpanzees 52.84 cm^2^) [82], rather they contain changes at all levels of organization that have been studied so far: the laminar and columnar structure of the cerebral cortex, the overall complexity of association areas of the cerebral cortex and their connections, especially long corticocortical pathways and the differences related to lateralization [83].

Furthermore, changes in neurophysiological processes, especially energy metabolism of the brain, occurred, but at a price [74,83]. Namely, an increase in aerobic metabolism leads to higher levels of oxidative stress, which favors the development of neurodegenerative diseases, and some of them can be seen only in humans, e.g., Alzheimer’s disease [74,84]. Comparative studies have shown that the brain of an adult at rest consumes 20–25% of the body’s metabolic energy, while other primates consume on average between 11 and 13%, and other mammals 2 to 8% [85]. This appears improbable, as it is a well-established physiological principle that larger organs and organisms consume less energy per unit of tissue than smaller ones (Kleiber’s law) [86]. Based on this principle, a larger human brain should expend less energy per gram of tissue compared to other primates, yet evidence reveals just the opposite: our brains are “running hot” [80,83]. In addition to the fact that transcriptome analyses indicate that approximately 74% of all human proteins are expressed in the brain and that 1,460 genes show increased levels of expression in the brain relative to other tissues, the importance of energy expenditure by glial cells, especially astrocytes, should not be forgotten [87]. Namely, in addition to ensuring the constant uptake of secreted neurotransmitters glutamate and γ-aminobutyric acid (GABA) from the synaptic clefts and by means of astrocyte-specific glutamine synthetase “returning” glutamine as a precursor of glutamate to neurons (glutamate/GABA-glutamate cycle, “glutamine-glutamate shuttle”) [88] thus preventing excitotoxicity mediated by excess glutamate, along with prevention of ammonia neurotoxicity [89], astrocytes also have a crucial role in the adequate supply of blood to the active parts of the brain since they are directly involved in neurovascular coupling [87] and cerebral autoregulation [90].

The enchanting cuteness that stems from neotenic qualities was probably first described by Konrad Lorenz [91], who suggested a set of traits (“*Kindchenschema*”) that make a creature “cute” and activate the motivation in another being to take care of it. In other words, infantile features trigger nurturing response in adults. People tend to react with caring behavior towards creatures that have the characteristics of neoteny such as a rounded head with relatively large eyes, while those with small eyes and long muzzles/beaks do not provoke an equally caring response [91]. For man, Lorenz said: “The number of enduring youthful traits of human beings is so great and so crucial to his overall behavior that I see no compelling reason to consider this universal youthfulness of man as nothing else other than true neoteny” [91]. More than 50 years later, functional magnetic resonance imaging (fMRI) has shown that neotenic qualities activate the brain’s reward system [92].

In most societies today, neoteny has become an imperative: for someone to look lovable and socially accepted, he must have the characteristics of neoteny. This is most evident today on social media and in the entertainment industry. In addition, cuteness is attributed not only to humans and animals, but also to objects or animated characters [93].

## 5. Willams-Beuren Syndrome and Schizophrenia

Insufficient knowledge on formation, migration, differentiation and proliferation of NCCs is perhaps best illustrated by the fact that disorders of these processes are considered as plausible underlying mechanisms of Williams-Beuren syndrome and schizophrenia, which are often classified as hyperdomestication disorders, as well as 7dupASD syndrome and autism spectrum disorders, which can be classified as hypodomestication disorders.

It is not uncommon for Williams-Beuren syndrome (WBS) to be also referred to as hyperdomestication syndrome, children with WBS being extremely obedient, sociable, and non-aggressive. People with WBS have also many other clinical features typical of domestication, such as delayed and reduced growth, shorter and wider noses, long philtrum, smaller skull and brain size, pointed ears, wide mouth and hypertelorism (large distance between the eyes), reduced size of upper and lower jaws, reduced teeth size, iris and skin depigmentation, increased basal oxytocin and vasopressin levels, slightly elevated levels of cortisol in stressful situations, as well as many other features corresponding to those seen in domesticated animals [94]. WBS is caused by hemideletion of about thirty genes on the long arm of chromosome 7 in the region 7q11.23 due to sporadic recombination in meiosis [95]. The functions of nine of the implicated genes (*GTF2I*, *LIMK1*, *ELN*, *GTF2IRD1*, *BAZ1B*, *STX1A*, *CLIP2*, *GTF2IRD1,* and *NCF1*) are relatively well-known.

The *GTF2I* gene (general transcription factor II-I) and its structural variants have been shown to be directly related to the tameness and social behavior of dogs [96] and red foxes [97]. In humans, this gene relates to the degree of social communication and anxiety [98] as well as the dorsolateral prefrontal cortex response to aversive stimuli [99]. The product of the *LIMK1* gene, LIM (short for the name of the three first discovered proteins from this group Lin11, Isl-1, and Mec-3) kinase 1 has a high degree of expression in the central nervous system. Hemizygosity for this gene in WBS is associated with impaired visuo-spatial abilities and reduced attention [100]. Due to mutations in the *ELN* gene for elastin, about 77% of patients with WBS have supravalvular aortic stenosis, probably owing to the fact that NCCs participate in the development of ascending aorta and arch of the aorta [101]. The genes *GTF2IRD1* [102] and *BAZ1B*, whose protein isoforms participate in chromatin remodeling and transcriptional regulation during induction and migration of NCCs, are considered the most important for cranio-facial features of WBS [103]. In addition, the hemizygosity of *GTF2IDR1* is responsible for reduced fear of strangers, which was also confirmed in the mouse model [104,105].

As already noted, many evolutionary biologists believe that the process of self-domestication is a consequence of community life and a reduction in evolutionary pressures for survival (relaxation of selection), resulting in a prolonged period of socialization. In such circumstances of the extended period of childhood, new social abilities and skills, especially those related to language, were developed [106,107]. Similar changes caused by domestication can be seen in different species today: it is known that in songbirds, the variability and complexity of singing increases with the length of domestication [108,109]. During this process, the lateralization of vocalization control in one hemisphere may occur. Nottebohm discovered in the 1970s that in canaries, finches, and sparrows, more than 90% of all singing produced through the vocal organ (*syrinx*) is controlled by the left hypoglossal nerve [110,111].

**Figure 7:**
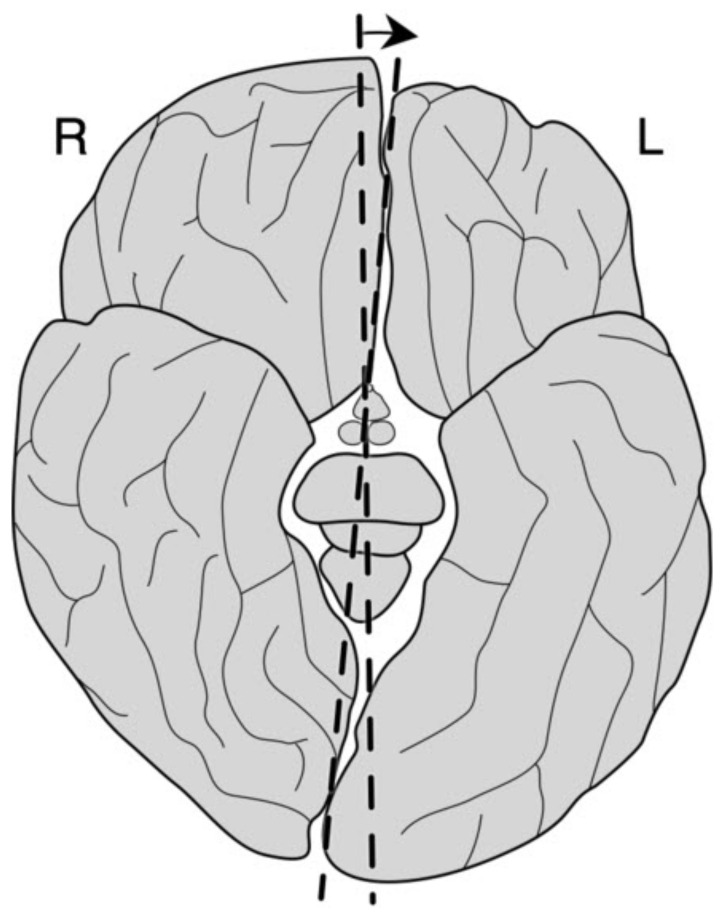
Illustration of the formation of asymmetry of the cerebral hemispheres. Since it was first described by Paul Yakovlev, it is also called Yakovlev’s twisting (torque) of the brain in a counterclockwise direction (Yakovlev cerebral anticlockwise torque). In the diagram, the twist is not shown from above, but from below (so in this view it is clockwise). Twisting also leads to asymmetry of the protrusion of the inner surface of the skull bones (petalia) as the bones adapt to the shape of the brain (and not vice versa). The scheme is from commons.wikimedia.org.

According to Crow’s hypothesis, schizophrenia arose sometime during the separation of man from other hominids (a “speciation event”), perhaps due to the transposition of part of the long arm of the X chromosome (Xq21.3) to the short arm of the Y chromosome [112]. This change was the basis for increase in the asymmetry of the cerebral hemispheres in order to concentrate communication and language abilities in one hemisphere (left), thus increasing the speed of processing and communication, hence also the probability of survival [113,114,115]. This process of lateralization of certain functions in the cerebral cortex, especially linguistic abilities, consisted of “moving/pushing” the structures around the lateral (Sylvian) sulcus of the right hemisphere forward. Therefore, in 97% of right-handed people, the frontal lobe of the right hemisphere is shallower and wider, whereas the frontal lobe of the left hemisphere is narrower and longer. Consequently, the parietal lobe of the left hemisphere is shorter, so the whole brain is torqued (Figure 7). Smaller or larger variations in the degree of lateralization are probably related to individual differences in the development of linguistic abilities. However, in individuals who do not develop typical cerebral hemisphere torsion [116,117] and associated predilection of the left hemisphere to develop a phonological component of verbal abilities [118,119], there is an increased risk of developing typical positive symptoms of schizophrenia, such as auditory hallucinations and persistent strange beliefs (delusions) [118]. This is supported by the finding of decreased temporal lobe volume in people with early-onset schizophrenia [120] and the finding that the brains of schizophrenics have a greater degree of symmetry or even reverse asymmetry [121]. A key component of the positive symptoms of schizophrenia are auditory hallucinations, especially voices heard only by the patient, which indicates a disorder of the indexicality mechanism by which we attribute and distinguish the voices we produce (or subvocalize) from those we hear from others. Such symptoms, in approximately 1% of the population, could be understood, according to Crow, as an evolutionary trade-off, which we as a species have opted for to develop language skills.

The description of the connection of certain physical characteristics with schizophrenia dates back to Hippocrates, and today it is well-established that patients with schizophrenia have more pronounced morphological (brachiocephalic head shape, facial asymmetry, ear shape abnormalities, reduced tooth size, oral anomalies, especially the palate), physiological (delayed onset of puberty [122,123,124,125,126], low testosterone levels, irregular menstrual cycle), neuroanatomical (reduced brain size [127], impaired development and structure of cortical columns) [128] and behavioral (abnormally low response to physiological stress) features of hyperdomestication [129,130]. This finding is not surprising since it is known that genes involved in neural crest development and domestication syndrome make up approximately 20% of all genes so far implicated in schizophrenia [107,129,131]. Perhaps the most difficult to explain is why people with schizophrenia have a slightly higher frequency of impulsivity and aggression, relative to the population average (which is a phenotypic feature opposite to domestication). One possible explanation is that it may not be constitutive, but in fact caused by long-term frustration of schizophrenic persons due to the lack of understanding from the people who surround them for their condition, as well as due to inability to control and plan wisely their own behavior [107]. Future studies of schizophrenia might focus more on genes essential for the development and migration of NCCs and domestication syndrome, as well as on the interaction of their products during normal and impaired development. For some of these genes, such as *FOXD3*, *RET*, *SOX9*, *SOX10*, and *GDNF*, their involvement in the evolution of language abilities is already well-established [107,132].

One of the potentially important new methodological approaches in the study of the neurodevelopmental theory of schizophrenia is the application of the concept of genomic regulatory blocks (GRBs). GRBs are chromosomal regions in vertebrates characterized by long, highly conserved (several hundred million years) non-coding sequences of DNA (conserved non-coding elements, CNE). Although some of them are very distant, most of these sequences serve as regulatory (activating or repressing) elements that act upon the promoter of a single target gene in the block region. This gene usually encodes a transcription factor (e.g., SOX9), and several transcription factors form a single regulatory network for a particular process during embryonic development and differentiation (see above). The usage of the GRB concept also contributes to a better elucidation of the biological effects of single nucleotide polymorphisms (SNPs), especially those found in non-coding gene regions (commonly in non-coding regions lay about 95% of hits obtained through whole genome-wide association studies, GWAS) [133,134]. Since the mid-1970s it has been known that non-coding elements have had crucial role in human evolution. Today, we are familiar with many congenital human diseases due to genetic changes (mutations, deletions, duplications, translocations) in CNEs, including Pierre Robin syndrome (brachydactyly-anonychia due to deletion/duplication of two CNE regions that regulate *SOX9* gene 1,450 and 1,200 kb from it, respectively), polydactyly/holoprosencephaly due to point mutations of two CNEs 1000 and 460 kb away from the *SHH* gene, respectively, aniridia due to CNE translocation 150 kb away from the *PAX6* gene, deafness due to CNE deletion 900 kb away from the *POU3F4*, brachydactyly A2 due to a CNE duplication 110 kb away from *BMP2* gene, Hirschsprung’s disease due to point mutation of intragenic enhancer of the *RET* gene, and many others.

## 6. Autism Spectrum Disorder (ASD)

Although this has not yet been fully confirmed, according to their phenotypic characteristics, 7dupASD syndrome and autism spectrum disorder (ASD) can be classified as hypodomestication disorders. 7dupASD syndrome (7q11.23 microduplication autistic spectrum disorder, Williams-Beuren region duplication syndrome, or Somerville-van der Aa syndrome) is caused by hemiduplication of genes in the same 7q11.23 region due to sporadic recombination in meiosis, and according to its clinical features (slow development of speech and language skills, mild facial dysmorphia, mild dysmetria and ataxia, difficulties in social interactions and communication skills such as autism spectrum disorders, social anxiety) [135,136] corresponds to hypodomestication. Slightly narrower forehead, thin lips, short philtrum, external ears rotated backwards, high palate, large teeth, and sometimes macrocephaly in children with 7dupASD are completely opposite to the appearance of children with WBS (caused by gene hemideletion in the 7q11. 23 region) who have a wider forehead, full lips, bitemporally narrowed cranium, short nose with a wide tip, long philtrum, less prominent zygomatic bones, prominent auricles, problems with strabismus and small teeth with large gaps in between them [135]. Also, relatively good visuospatial abilities (but with poor eye contact), normal response to loud sounds, difficulties in articulation and communication (including poor use of gestures and facial expressions) in children with 7dupASD are in sharp contrast to poor visuospatial abilities, hypersensitivity to sound, language fluency and hypersociality of children with WBS [135]. Hemiduplication of the *GDF2I* gene is thought to be responsible for most features of 7dupASD [137].

Autism spectrum disorder as a “new form of emotional disorder” and an “anxiously obsessive desire to maintain monotony” was first described in eleven children by Kanner in 1943 [138,139]. Even then he assumed that the cause of the disease was genetic [138]. According to the fifth edition of the Diagnostic and Statistical Manual of Mental Disorders of the American Psychiatric Association (Diagnostic and Statistical Manual 5, DSM-5) from 2013 [140], to make a diagnosis of ASD it is necessary to meet all three criteria from category A—social interaction and communication (1. existence of deficit in socio-emotional relationships with a reduced degree of sharing interests, emotional and affective experience with other people, as well as the inability to initiate or respond to social interactions, 2. the existence of deficits in nonverbal communication such as eye contact during conversation, use of facial expressions and gestures, 3. the existence of deficits in developing, maintaining, and understanding relationships with other people together with behavioral adjustment deficits in different social contexts with lack of interest and sharing their own ideas with peers) and two of the four criteria from group B—limited and repetitive behaviors, interests and activities (1. performing stereotypical or repetitive motor patterns, 2. insisting on monotony and inflexible attachment to everyday routines or rituals [e.g., constantly eating the same food, experiencing extreme stress due to small changes in the environment, rigid thought patterns], 3. highly limited and fixed interests [e.g., high degree of preoccupation and attachment to unusual, but same objects], 4. hyper- or hyporeactivity to sensory stimuli [e.g., indifference to pain or temperature changes, hostile response to specific sounds or textures of objects, excessive touching or sniffing of objects, excessive fascination with lights or movements]). ASD occurs more frequently in boys than in girls (approximately 4-5:1) [141]. Since the 1980s, the incidence of ASD has been steadily increasing. For example, in the 10-year period from 2000 to 2010, the estimated incidence of ASD increased by 119.4% (from 1:150 to 1:68 births), and in 2016 it was 1:40, with a prevalence of 2.5% (3.63% for boys and 1.25% for girls) [142].

**Figure 8:**
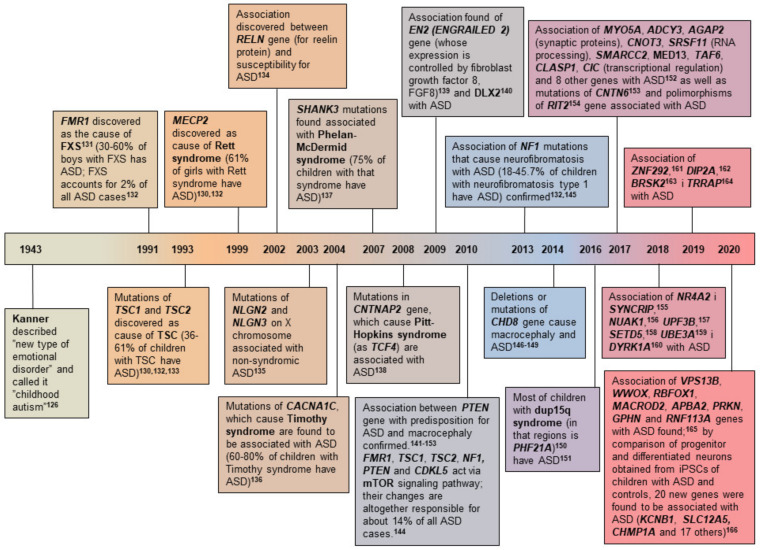
Time-lapse of major discoveries in regard to genes associated with autism spectrum disorder (ASD). FXS, Fragile X syndrome; iPSC, induced pluripotent stem cells; TSC, tuberous sclerosis complex. See text for details.

Thanks to clinical, genetic, neuropathological and neuroimaging research, understanding of the etiopathogenesis of ASD has been steadily growing since Kanner’s time, but the key molecular pathways that lead to ASD are still unknown [143]. The most likely reason for this is great heterogeneity of factors, primarily genetic but also environmental factors that may be involved in the development of ASD [144,145]. The clinical picture is also variable and can differ dramatically even in identical twins [146]. Based on the clinical picture ASD can be divided into syndromic ASD and nonsyndromic ASD [143]. More than 70% of children with ASD have syndromic ASD, which means that in addition to the symptoms of ASD, they also have various comorbidities such as reduced intellectual abilities, speech and language deficits, epilepsy, motor and morphological abnormalities, gastrointestinal difficulties, and others [143,147]. Figure 8 shows sequence of important discoveries of individual genes associated with ASD.

In addition to the fact that only sometimes the so-called common sequence variants (meaning that the minor allele frequency [MAF] is greater than 5% in the population) or their combinations are implied in the development of ASD, more important for the pathogenesis of ASD are rare (MAF < 1%) or very rare (MAF < 0.1%) single nucleotide variants (SNVs), as well as rare variations in the number of copies (copy number variation, CNV), especially deletions, but also duplications [148,149,150,151,152]. As of April 20, 2020, the Simons Foundation Autism Research Initiative (SFARI), the largest international database for ASD researchers, had a total of 835 genes whose changes or variants are associated with ASD (https://gene.sfari.org/). All genes are divided into three groups: in the first group are those with high reliability that their changes or variants are associated with the emergence of ASD (Score 1—high confidence genes, about 150 genes). About one third of genes in this group (52 genes) are also involved in the formation, migration, or differentiation of NCCs. The second group contains genes for which there are findings that suggest that they could have an impact on the development of ASD (Score 2—strong candidate genes, about 220 genes), while the third group contains genes for which there are results that suggest that they could be involved in the pathogenesis of ASD (Score 3—suggestive evidence genes, about 470 genes).

Regardless of the group in which they are located, those genes whose changes or variants are known to cause disorders other than ASD are also called syndromic ASD genes. For example, syndromic ASD genes include: *ANKS1B, ARID2, **ATP1A1**, **ATP1A3**, **CACNA1C**, CAMK2A, CEP290, **CHD1**, CLCN4, CNKSR2, **CNTNAP2**, CSDE1, CUX2, DEAF1, **DLL1**, DOLK, FBX011, **FMR1**, GABBR2, **HDAC4**, **KAT6A**, KIF5C, KMT2E, MED12L, **MEF2C**, **MTOR**, NACC1, **NF1**, **NFIB**, **NR2F1**, NR3C2, NTNG1, PPM1D, POGZ, **PRODH**, **PTEN**, RNF135, RPS6KA3, **SATB2**, SCN1A, SETD1B, SHANK3, SMC3, SYNE1, TCF4, TRAF7, TRRAP, TTN, UBE3A, UNC13A, and USP7*. The bolded genes in this list are involved in the formation, migration, or differentiation of NCCs. A large number of genes involved in the pathogenesis of ASD are associated with synaptic transmission (e.g., *FMR1, SHANK2, SHANK3, NLGN2, NLGN3, CACNA1C, EN2, NRXN1, NRXN2, NRXN3, SCN1A, SCN2A*, and many others) as well as genes whose protein products are involved in the regulation of transcription and chromatin remodeling (e.g., *UBE3A, MECP2, CREBBP, EP300, PCDH19, NCKAP1, ZNF292, ZNF462, BAZ2B, CHD7, CHD8,* and others).

An excellent example of a syndromic ASD gene is *FMR1* (fragile X chromosome and mental retardation 1). Expansion of the CGG nucleotide triplets within the *FMR1* gene located on the X chromosome results in increased sensitivity of the gene to epigenetic silencing by methylation. In healthy individuals, there are between 5 and 44 CGG repeats, 45-54 repeats form a “transition zone,” and 55–200 repeats are considered premutation [153]. *FMR1* gene premutation is responsible for three groups of disorders associated with the fragile X chromosome: 1) tremor and ataxia syndrome (fragile X-primary tremor/ataxia syndrome, FXTAS) [154], 2) fragile X primary ovary insufficiency (FXPOI)—approximately 20% of women with premutation have this disorder and enter menopause before the age of 40, and all are significantly more likely to have children with fragile X syndrome (FXS) and 3) fragile X-associated neuropsychiatric disorders (FXAND). At full mutation, more than 200 repeats are found in FXS, resulting in loss of gene expression. The main symptoms of FXS include slow development of language skills, mild to moderate intellectual difficulties, and difficulties in social interactions, hyperactivity (sometimes in the form of attention deficit hyperactivity disorder, ADHD), and about 10% of them have epileptic seizures. The incidence of FXS is twice as common in boys (occurs in approximately 1 in 4,000 newborns) than in girls (1: 8,000). Due to the compensatory action of the *FMR1* gene from the second X chromosome, affected girls usually have a milder clinical phenotype than boys. The physical appearance is characterized by a pronounced jaw and forehead, elongated face and flexible fingers, and after puberty boys have testicles significantly larger than average (macroorchidism). About 30–60% of boys with FXS have ASD [144,146,155].

Another good example of the influence of epigenetic changes on the development of ASD-like symptoms is Rett syndrome. It was first described by Rett in 1966 [156]. Its key features are ASD-like symptoms: intense screaming, relentless crying, avoidance of eye contact, lack of social and emotional communication, disturbed nonverbal behaviors that regulate social interactions, absence of speech or speech disorder, sensory disturbances, sleep disorders, epilepsy, difficulties in movement coordination, repetitive hand movements, and others. Rett syndrome is caused by mutations in the *MECP2* gene whose protein product methyl-CpG-binding protein 2 (MeCP2) is one of the key mediators of epigenetic regulation as it regulates the expression of a large number of genes [157,158,159]. Although in vitro experiments showed that MECP2 is a transcriptional repressor protein, in vivo experiments yielded surprising results in regard to the abundance of genes affected by MECP2: in the hypothalamus of 6-week-old mice without *Mecp2* genes and transgenic mice of the same age with the added *Mecp2* gene, there was a disrupted expression of 2184 and 2582 genes, respectively [159]. Compared to controls, in the model without the *Mecp2* gene, about 85% of the genes had reduced expression levels and about 15% elevated, and in the model with the added *Mecp2* gene it was the other way around: about 85% of the genes had an elevated level of expression and about 15% of the genes had decreased levels of expression [159,160,161,162,163,164,165]. Today, it is known that the MeCP2 protein acts through at least 13 different partners that can be classified into three groups [166]. The first group comprises histone deacetylases (histone deacetylases, HDAC) that contain NCoR1/2 and SIN3A complexes and locally deacetylate lysine residues on histone tails, resulting in the transformation of chromatin into a more condensed form. The second group includes transcription factors YY1, SOX2 and SP3, and the third group includes histone methyltransferases PRMT6, G9a or HLCS, as well as chromatin-transforming BRM protein [166]. Recent research shows that MeCP2 protein deficiency in Rett disease does not only affect neurons, but also glial cells, especially astrocytes (increase in extracellular glutamate concentration is seen due to decreased expression of excitatory amino acid transporter 1 (EAAT1) and increased expression of glutamine synthetase, as well as increased expression of glial acid fibrillar protein [GFAP] and S100 protein) and oligodentrocytes (thinner myelin due to reduced expression of genes associated with myelin production) [167]. Rett syndrome is rarely inherited (< 1% of all cases) as it usually occurs as a new mutation in one in 8,500 newborn female children (boys usually die shortly after birth). According to the 2013 DMS-5 criteria, Rett syndrome no longer belongs to the group of autistic disorders, but it has been singled out as a separate disease [140,168]. In contrast, Asperger’s syndrome (for which the criteria are met by people with ASD who speak fluently, although still have problems related to verbal communication and social communication in general, e.g., speak in a monotone voice, have unusual vocal expression and choose unusual topics for conversation, and have average or above-average intelligence) and pervasive developmental disorder (PDD), that were considered as separate disorders before 2013, nowadays fall under ASD umbrella [140].

Yet another example of the influence of epigenetic changes on the development of syndromic ASD is the duplication of q11.2-13.3 regions on chromosome 15 (dup15q syndrome) [164]. Namely, duplication of maternal chromosome 15 is one of the most common individual changes associated with ASD, while duplication of the same area of the paternal chromosome 15 almost never lead to ASD [169]. This is an interesting fact because it suggests that studying the effect of maternal genes on that locus, especially *UBE3A*, could serve to better understand the pathogenesis of ASD. Deletion of genes from this region inherited from the father (*MKRN3*, *MAGEL2*, *NDN*, *SNURF-SNPRN* genes) leads to Prader-Willi syndrome (PWS) [170,171].

Deletion is responsible for about 70% of all PWS cases, while in 25–28% of cases PWS occurs due to inheritance of both homologues from the mother (maternal uniparental disomy, which is the functional equivalent of deletion of that part of chromosome 15 from the father). In 2–5% of cases, the cause of the disease is mutations in the imprinting center.

Children with PWS have pronounced central-type hypotonia, which means that there is a problem above the level of the lower motoneuron. Difficulty feeding in the newborn leads to hyperphagia around the second year of life and frequent obesity later on. Children with PWS also have symptoms of hypogonadism—general developmental delay, short stature, small hands and feet, and genital hypoplasia. Most of them also have mild features of dysmorphia—elongated eyes, narrow forehead, and triangular upper lip. Almost all have pale skin and hair, mild to moderate intellectual disabilities, as well as behavioral and articulation problems. ASD has been documented in 38% of PWS cases due to maternal uniparental disomy, and in 18% of patients with deletion [172,173].

The lack or absence of maternal gene expression in the same region (of *UBE3A* and *ATP10C* / *ATP10A*) causes Angelman syndrome (AS) [174]. In most cases (65–70%) it is a *de novo* deletion, and in a small number of cases, the disease is caused by the father’s uniparental disomy (which is the functional equivalent of deletion of that part of maternal chromosome 15) [175]. The rarest cases are those with abnormal methylation of that part of the 15th maternal chromosome, or with mutations within the mother’s *UBE3A* gene [175]. Children with AS have developmental delays, microcephaly, a wide mouth, protruding tongue, mandibular prognathism, and epileptic seizures, as well as ataxia and tremor with poor speech development. Nevertheless, they are always characteristically cheerful and smiling [174], and ASD is seen in about 2% of cases [172,173]. These differences between PWS and AS were the first evidence of genomic imprinting.

Thus, the loss of expression of *UBE3A* causes AS, while its duplication leads to ASD [176,177,178,179]. Duplications affecting only the 15q11.2 or 15q13.3 locus are different from those of the entire area 15q11.2-q13.3 [179]. People with 15q11.2-q13.3 duplication, i.e., with the dup15q syndrome, have some features of PWS and AS, but also some unique features, such as sensory perception disturbances [179,180]. Most of them meet the criteria for ASD (avoidance of eye and physical contact, poor social skills, speech and language retardation, and stereotypical behaviors [biting nails, spinning in a circle, etc.], many also never adopt a symbolic game).

There are two forms of chromosome 15 duplication: the more common form is the isodicentric chromosome 15q, which means that in addition to the two normal copies of chromosome 15 there is one small redundant chromosome with two additional copies of the maternal 15q11-2-13.3 area, meaning three maternal copies of the locus and one paternal (where greater number of the maternal copies means higher probability for ASD) [181]. A less common form is maternal interstitial duplication, meaning that there are two maternal copies of the locus and one paternal (there are also individuals with two paternal locus duplications, but they are less common and have a milder phenotype) [169]. Of the several genes in this locus, two maternal genes appear to be particularly important for the pathogenesis of the disease: *UBE3A* encoding ubiquitin protein ligase E3A and *ATP10A* (ATPase phospholipid transporting 10A) encoding the transporting phospholipid ATPase. Duplicated segment also includes two paternal genes that may be involved in the development of ASD: *MAGEL2* (MAGE family member L2) and *NECDIN* (*NDN*). As this group of genes also contains genes encoding the γ-aminobutyric acid receptor subunits (*GABRB3*, *GABRA5*, and *GABRG3*), as well as the gene for another ubiquitin ligase (*HERC2*), it is believed that these genes, especially *GABRB3*, may also contribute to development of ASD [169,182]. Maternal interstitial duplications account for 1–3% of all non-syndromic (idiopathic) cases of ASD [183].

In addition to mutations and changes in genes associated with non-syndromic ASD and mutations and changes in genes that in themselves cause a pathology that may or may not be associated with ASD, some of the presumed risk factors for autism also include: (1) low birth weight (head circumference at birth is lower than average, see later), (2) exposure to heavy metals and environmental toxins and pollutants in the air [184,185], which can lead to decreased expression of *RELN* gene and consequently to abnormal positioning of cortical neurons (the product of *RELN* gene – protein reelin serves as signal for stopping radially migrating neurons into the cortical plate [186,187], whereas *RELN* gene promoter is hypermethylated in persons with schizophrenia in comparison to controls – together with concomitantly increased expression of DNA methyltransferase 1 (*DNMT1*) [188], so some authors suggested reelin measurement in blood as a possible biological marker of autism [189]), (3) maternal exposure to xenobiotics and endocrine disruptors during pregnancy [190,191], (4) viral infections such as rubella [192], (5) fetal exposure to some drugs such as valproic acid and thalidomide, (6) various metabolic disorders including vitamin D deficiency [193,194], maternal thyroid dysfunction (especially hypothyroidism) [195], as well as many others.

**Figure 9:**
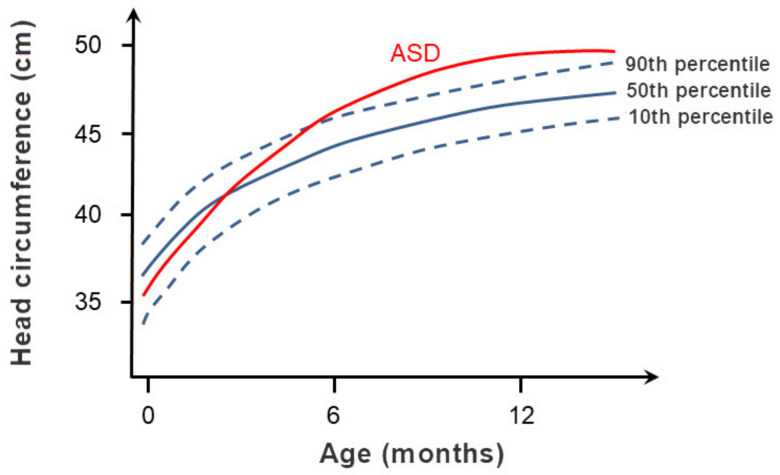
Early excessive growth of head circumference in children with autism spectrum disorders (ASD). After reduced head circumference at birth, during the first year of life in children with ASD there is a pathologically increased growth in head circumference and the total size of the cerebellum and cerebellum. The graph is based on Courchesne et al. (2003) [196].

Apart from genetic and epigenetic, other changes in ASD can be divided into morphological-neuroanatomical, neuroimaging, and biochemical. The most conspicuous morphological change is early overgrowth of the brain in the first year of life (Figure 9) [196].

After the age of 3, or perhaps even earlier, the size of cerebellum in children with ASD is also, on average, proportionally larger than in controls [197]. Although total hemisphere volume is larger, vermis size and the number of Purkinje neurons in the lateral parts of the hemispheres are reduced [197,198]. This is important, as the role of the cerebellum in both cognitive and emotional processing is well-established [197]. In the context of cerebral changes, it should be noted that in children with ASD, amygdala volume is also increased [199,200], and the degree of this increase correlates with the severity of social and communication deficits [201,202] and anxiety [203]. For example, according to a study of 45 children with ASD aged 36–56 months using 1.5 T magnetic resonance imaging, this group of children had a significantly higher average amygdala volume compared to the control group of 26 children. The difference was 13.6% increase for the left amygdala and 16.6% increase for the amygdala in the right hemisphere [204]. The association of higher amygdala volume with communication deficits is not surprising, as an earlier study already documented that children with greater amygdala volume had more speech and language difficulties [205].

In contrast to increased volume, in all amygdala nuclei, a reduced number of neurons was documented using the unbiased stereological fractionator method both in children (10 children with ASD and 7 controls aged 2 to 13 years) age) and adolescents (6 ASD and 5 control subjects aged 14 to 20 years) as well as in adults (9 ASD and 10 control subjects older than 21 years) [206]. This finding is consistent with decreased emotional capacity and increased susceptibility to psychiatric disorders (eating disorders, depression, and anxiety disorders, especially obsessive-compulsive disorder) in people with ASD [207]. In contrast, the increased number of modified pyramidal neurons (von Economo neurons) in layer 5 of the frontoinsular cerebral cortex in people with ASD may be related to the clinical observation of increased interception of people with ASD (higher relative focus on intrapsychic events in relation to environmental stimuli) [207].

Another notable neuroanatomical change in ASD is the excessive number of cortical columns and the greater degree of dispersion of neurons in the frontal and temporal lobes and anterior cingulate cortex [208,209]. For comparison, there is no evidence that the columnar microarchitecture is affected in e.g., visual cortex whose development ends in the first few months of life [208], leading to the conclusion that those regions of the cerebral cortex whose development takes the longest are more severely affected in ASD. The human cerebral cortex contains approximately 70–80% of excitatory pyramidal neurons and 20–30% of inhibitory interneurons. Nearly one-fifth of inhibitory neurons arrives into the cerebral cortex through a complex process of tangential migration from the ganglionic eminence [210]. Less developed inhibitory interneurons, such as basket and chandelier neurons, together with dysfunction of certain structural synaptic proteins (neurexin, neuroligin, PSD-95, SHANK3, contactin, and others) are believed to lead to an increased excitation-to-inhibition ratio [211] and contribute to an increased susceptibility of people with ASD to epileptic seizures [212]. Epileptic seizures occur in about one-third of people with ASD, while in about 60% epileptiform activity can be recorded during sleep [212]. Epilepsy and its consequences are among the most common single immediate causes of death in people with ASD [213].

A pronounced feature of children with ASD is weaker functional connectivity of the left and right hemisphere. In one study, cerebral cortex activity was recorded in children aged 12–46 months (mean age 29 months) during normal sleep (without sedation) by sound stimulation for 20 to 30 s [214]. Sound stimulation contained words, sentences, ambient sounds (train, telephone, airplane, dog barking), and the values for each voxel were obtained by subtracting the measured value of the blood oxygenation level-dependent signal (BOLD) from the value of the same signal over a period of equal duration in which there was no stimulation [214]. On average, in children with ASD, weaker interhemispheric synchronization in linguistic areas was statistically significant as early as 14 months of age [214]. Other studies have also shown that ASD brain lacks typical lateralization in which the left hemisphere is dominant for speech-linguistic abilities. Quite opposite, in some ASD brains, even the reverse lateralization can be seen. For example, when the activity of the cerebral cortex during speech was observed in ASD children aged two and three years, a significantly higher activation of the right hemisphere was found both in relation to the control group of children matched for age and in relation to the control group of children matched for mental abilities [215]. These results were confirmed another cohort of children with ASD [216]. Since these studies have shown that in children with ASD the right parietal lobe deals with the processing of language content, this consequently implies difficulties in maintaining attention and emotional processing, since in children who develop normally these processes take place almost exclusively in the right hemisphere. Reduced degree of asymmetry in cerebral cortex thickness in a total of 1774 children with ASD compared with 1809 control peers from 54 independent data sources was confirmed for medial part of the frontal lobe, orbitofrontal cortex, cingulum and lower part of the parietal cortex [217].

**Figure 10:**
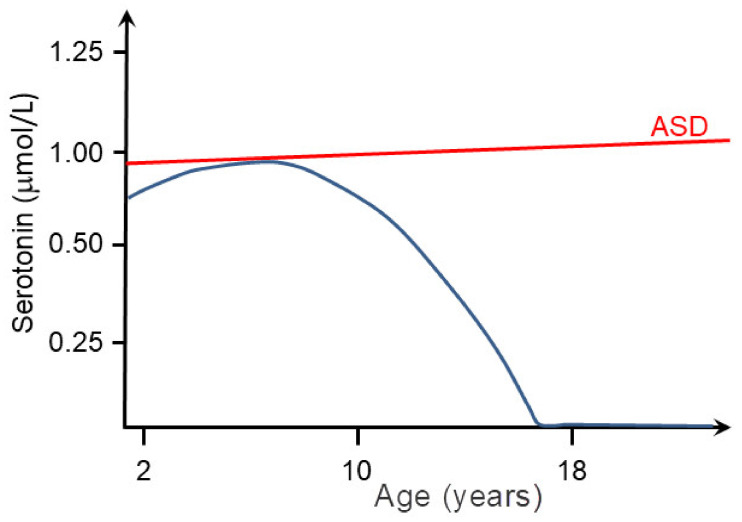
Schematic representation of increased serotonin levels in the blood (hyperserotonemia) in individuals with autism spectrum disorder (ASD). The blue curve presents the concentration of serotonin in the blood in control subjects. Graph based on Laboyer et al. (1999) [219].

One of the earliest discovered abnormalities among the biochemical changes in ASD is impaired serotonin transmission [218]. Increased serotonin levels in the blood (age-independent hyperserotonemia) are present in approximately one-third to one-half of people with ASD (Figure 10) [143,212,219,220]. Disorders in serotonin neurotransmission lead to learning difficulties, especially in relation to the development of speech and verbal abilities [220] and generally have negative effect on cognitive abilities and behavior (self-harm, anxiety, depression, aggression, antisocial behavior). Since serotonin mediates a number of developmental processes, from neurogenesis, cortical morphogenesis, cell migration, and synaptogenesis [221,222], to synaptic plasticity, proinflammatory cytokine secretion by immunocompetent cells, and volume transmission [223], it is possible that changes in genes involved in the serotonin system, especially *MAOA* (monoamine oxidase A), *MAOB* (monoamine oxidase B) and *SLC6A4* (serotonin transporter) lead to abnormal neural circuits in ASD [212]. Serotonin blood levels are most affected by its production in enterochromaffin cells, but also by changes and variants of the *ITGB3* gene encoding β3 subunit of integrin, a protein involved in a transporter complex that carry serotonin into platelets [224]. It is believed that hyperserotonemia in ASD is not associated with the central serotoninergic system, which critically depends on the expression of *TPH2* gene [225].

Considering that compared to extinct hominins, modern human has had pronounced features of the domestication syndrome for at least 50,000 years (reduced brain size, reduced degree of aggression, neoteny and others), it can be concluded that these features are generally contrary to the known features of ASD. Therefore, it can be concluded that a significant part of pathophysiological processes, as well as the clinical presentation of ASD, may arise due to reduced neural crest cell function. If confirmed, this would classify ASD in the group of hypodomestication disorders. Some of the prominent features of ASD as a hypodomestication disorder are the following: (1) morphological changes of the cranium: excessive skull growth (macrocephaly) and higher brain volume in early childhood, including, on average, higher volumes of cerebrum, cerebellum, hippocampus, and amygdala in individuals with ASD compared to control subjects with typical development, (2) morphological changes of the face, teeth, and ears, (3) increased and prolonged elevated cortisol levels upon stressful stimulation and a pattern of cortisol secretion that is characteristically higher toward the end of the day instead in the morning, (4) increased adrenocorticotropic hormone (ACTH) levels that correlate with the severity of symptoms, (5) overall poor adaptability and physiological response to psychosocial stress stimuli: hyperreactivity of the hypothalamic-pituitary-adrenal axis to mildly unpleasant stimuli and relative hyporeactivity in threatening social situations with real danger, (6) features opposite to neoteny: increased androgen levels in the blood regardless of age, faster postnatal growth and a higher degree of irritability and aggression; in about a quarter of people with ASD aggression is significantly higher than average, although it is more reactive (latent) than proactive type of aggression, typically expressed as irritability that correlates with poorer adaptability, degree of social deficits, and weaker verbal abilities (i.e., overall severity of the clinical picture of ASD), (7) delayed onset of menarche (first menstruation), 8) reverse lateralization of cerebral hemispheres (possibly affected by elevated concentration of testosterone) with poor interhemispheric connectivity [226].

The assumption that ASD is hypodomestic syndrome is supported by a long list of genes responsible for certain endophenotypes of ASD that are known to be important in the development of NCCs and their derivatives. According to the gene ontology classification, these genes mainly belong to the following groups: metabolic processes (about 22% of genes), biological regulation (about 19%), developmental processes (about 17%), cellular processes (about 14%), processes related to intercellular communication (about 12%), immune processes (about 4%), and apoptosis (about 3%) [226,227]. According to signaling pathways, most of them are involved in axon guidance (about 12% of genes), signaling pathways of the transforming growth factor-β, TGF-β, about 10%), endothelin signaling pathway, gonadotropins-releasing hormone signaling pathways (about 8%), WNT signaling pathway (about 8%), FGF signaling pathway (about 8%), angiogenesis (6%) and others [228].

The finding that signaling pathways associated with axon guidance are the most common feature of documented changes in ASD is not surprising, because comparative genetic analyses consistently show that changes in the degree of brain myelination, especially of the frontal lobe, have begun as early as during the period of existence of common (now extinct) ancestors of humans and chimps and continued even more after split of human from other hominins. This has been confirmed by comparison of human, chimp and rhesus transcriptome in the prefrontal cortex [229]. That analysis showed that oligodendrocytes have far more differentially expressed genes than neurons, especially those genes for which GWAS studies showed significantly different expression in ASD, schizophrenia, ADHD, major depressive disorder, and bipolar disorder [230].

In regard to aggressive behavior, it should be noted that aggressive behavior at certain stages of development is a part of the typical repertoire of children’s behavior. Children usually show the highest degree of aggression between 2 and 3 years of age. The reason for this is thought to be their need for increasing autonomy and lack of inhibitory control due to the slow maturation of neural circuits involving the orbitofrontal and prefrontal cortex. As, at that age, children still do not have well-developed speech skills and cannot communicate well enough about what bothers them, they use aggression as a means of expressing their own needs and a way of dealing with anxiety and irritability. However, with increasing mastery of communication skills, aggressive behavior decreases during a child’s typical development. This is not usually the case in children with ASD, and a persistent lack of inhibitory control is statistically significant even when the influence of attention is removed as a possible confounding variable (variable of indirect influence) [221]. Although according to the DSM-5 criteria, language deficits in ASD are no longer considered as a central feature of the disease (as they are now classified under communication problems). Also, considering the difficult examination of speech-language development due to its masking by other deficits and symptoms of ASD, the information presented here suggests that genetic changes seen in ASD could represent one of the evolutionary diversions, or “wrong directions” of ontogenetic development of language skills that to a greater or lesser extent arise from changes in genes important for the domestication syndrome, i.e., genes that mediated self-domestication through control of neural crest cell derivatives.

Another speculation is that only a part of the human social evolution could be related to self-domestication (the one that we share with other mammals), whereas other unique aspects of mimetic and language communication in humans, which required socially-mediated emotional plasticity accompanied by new social emotions driven by culturally-learned emotional control, thus challenging the notion of human self-domestication in favor of self-control [231].

Finally, while most researchers would probably agree that domestication can be regarded as part of evolution, some authors see domestication “formation” as a process somehow different from evolution. This thought was coined by Bohlken in 1961 [232] and later by Herre and Röhrs in 1990 [233]. It was summarized and developed further by Zeller and Göttert [234]. In their own words:

“We think that the process of domestication, however, is fundamentally different (from evolution, authors’ comment): separation of a species from its natural ecological context, development of a form of this species via artificial selection (mostly eliminating natural selection, loss in genetic diversity) in an artificial environment that is dramatically different from the natural environment. In short: the nature of the domestication process appears as an attempt to minimize the driving forces of evolution as far as possible. Not surprisingly, the rules and laws of population ecology and population genetics are no longer applicable to domesticated forms (see temporal dimension, and numerical dimension).”

(page 8, lines 4-9)

## 7. Evolutionary Importance of Emotions

Charles Darwin was the first to investigate the evolution of emotional reactions and facial expressions. He described his thoughts in the book “*Expression of emotions in humans and animals*” [229]. Darwin recognized the importance of emotions for the adaptation of the organism to various stimuli and situations in the environment. Modern views define emotion in such a way that each individual emotion has evolved through evolutionary history by some kind of natural selection, i.e., by expanding the repertoire of behavior that leads to coordinated cognitive, physiological, and behavioral responses that increase the likelihood of survival and reproduction. Negative emotions (of fear, anxiety, pain, sadness, jealousy, grief, anger, guilt, shame) are mainly associated with increased likelihood of survival, while positive emotions are mostly associated with increased reproductive success and care for offspring (excitement, joy, pleasure, desire, hope, infatuation, pride, optimism, friendship, love) [235,236].

The adaptive value of negative emotions consists in motivating the organism to avoid potential injury or death by escaping from a dangerous situation. These emotions can also serve to prevent damage, to attack (counterattack) or to repair damage. The emotion of fear motivates organism to escape from a dangerous situation, the emotion of panic indicates an immediate attack by predators, anger motivates to attack, disgust motivates avoidance (or vomiting in case of possible poisoning), general anxiety indicates an insecure environment, separation anxiety warns about possible separation from protective parent(s), anxiety from strangers about the possible harm they can inflict, obsessive cleanliness indicates increased fear of infectious diseases, obsessive hoarding indicates fear of losing food or other resources, agoraphobia is a warning sign of an environment where the likelihood of an attack is high, phobias of small animals indicate the possible danger they may cause, hypochondria may indicate a false alarm or situation in which one might become ill, fear of blood a situation in which injury may occur, emotion of personal inappropriateness a situation of rejection by an ally or friend, while emotion of sadness and depression motivates to seek help or give up potentially futile plans. Although negative emotions are extremely adaptive, in excess they can cause serious emotional disorders such as anxiety and depression.

An individual who recognizes and responds to hints of threats from the environment lives longer and has more offspring than those who have to face a real threat [237]. Therefore, negative emotions such as anxiety and low mood (depression) should not automatically be considered as disorders as they have adaptive value. To define the boundary between physiological levels of negative emotions and pathology that needs to be treated is therefore not an easy task to do, but at least to some extent we should embrace more the fact that negative emotions have evolved as our defense mechanisms, such as pain sensation [237]. Consequently, it is not uncommon for evolution to “like” variants of genes that promote anxiety because it is better to have more false positive responses than just a single false negative (type 2 error), which can endanger existence. Emotional disorders often have their origin in social emotions as conflicts are an unavoidable part of social life. Like anxiety, depression has also a positive adaptive value (if the processes underlying these two disorders had no adaptive value, their prevalence would not be so great today). In evolutionary terms, depression helps body to cope with unfavorable situations because it prevents dangerous or useless actions in situations when the organism strives for a goal that it cannot achieve (e.g., to fight an opponent when injured). Deeper and analytical thinking requires isolation and a high degree of concentration, so depressed people tend to be lonely and intensively consider their own problems (rumination). Studies of the brain activity of depressed people and controls using fMRI confirmed that depression is dominated by introspection, mainly mediated by an increased activity of the default mode network (DMN) [238,239].

At first glance, the evolutionary importance of positive emotions such as joy, serenity, or gratitude does not seem as great and obvious as that of negative emotions necessary for survival and adjustment [240]. Yet today, positive emotions allow people to have a greater repertoire of thoughts and actions, which leads to greater mental flexibility, optimism and social connections (strengthening existing connections and creating new ones). Consequently, positive emotions have a beneficial effect on health and longevity, development of competencies and skills, motor coordination and strength, ability to learn new information and solve problems [240,241]. Positive emotions and playfulness are associated with increased reproductive success, but also with increased resilience to difficult circumstances, a sense of belonging and identity development, as well as purposeful, goal-oriented behavior. Finally, because of the positive emotions we have toward our children, family, and members of the immediate and wider community, we are willing to even endanger our own lives to pass on our genes to the next generation [240].

The effects of reciprocal emotional exchange are at the heart of evolutionary research into human social behavior and social neuroscience [235,242]. In order to study these interactions, mathematical models have been developed, which are collectively known as “game theory”. The concept was developed to understand a wide range of social interactions, including competition, collaboration, and coordination. The game involves two or more people, thus providing a basis for linking individual decisions to group’s outcome. The prisoner’s dilemma is a canonical example of such a game through which the balance between personal and group benefits can be studied, that is, between competition and cooperation in business, politics, or any other social environment [242]. The state of equilibrium in any of the studied models (games) in which each player has chosen his strategy of play and none can profit from a change of strategy as long as other players use their strategies is called Nash equilibrium [242,243].

**Figure 11:**
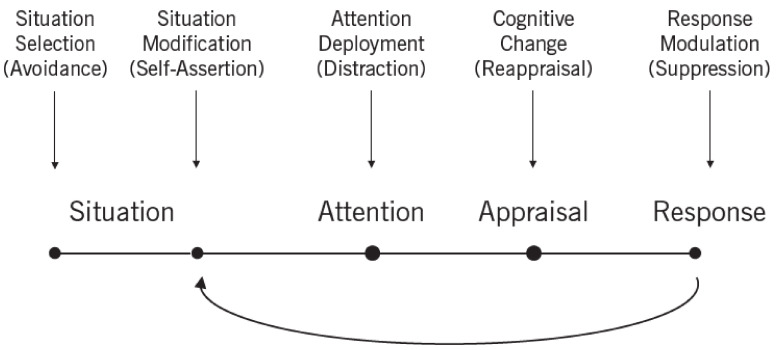
Schematic representation of the five-point model of processes involved in the regulation of emotions according to Gross. For any emotional event, there are a number of steps on which emotion regulation can be applied. See text for details. Slightly modified from Gross (2013) [244].

An extremely important aspect of emotions is the possibility of their control, which can be significantly disturbed in various states, for example in mood disorders. The term emotion regulation is generally used to describe conscious attempts to manage emotions (which of course does not mean that regulation does not take place on an unconscious level as well) in such a way that they are socially acceptable and not “out of control”. This regulation can be applied to the various steps involved in emotional experience (Figure 11) [244,245].

Avoiding social situations for the sake of emotional regulation is especially pronounced in people with social anxiety disorder and avoidant personality disorder. Situation modification refers to changes in the external environment, such as an increase in physical distance from another person. Attention deployment can be (1) passive and repetitive focusing on symptoms and consequences of stress (rumination), and often occurs in major depressive disorder, (2) it may involve directing attention to potentially negative events in the future (worry, anxiety), which is one of the most common symptoms present in anxiety disorders, especially generalized anxiety disorder, (3) it may involve suppression of thought, which may temporarily lead to emotional relief, but in the long run actually encourages unwanted thoughts and results in obsessive-compulsive disorder. Rumination, anxiety, and suppression of thoughts are considered inappropriate forms of emotion regulation. Cognitive change of assessment includes reassessment/reappraisal (e.g., reinterpretation of the situation and perception of the “bigger picture”), distancing (perception of the situation from a third person perspective) and healthy humor. These are the most appropriate and effective ways of emotion regulation. Adjustment of emotional responses involves their suppression, which correlates with difficulty building relationships with other people and other negative social consequences, including the use of addictive substances, which is considered an inappropriate form of emotion regulation. On the other hand, exercise and increased sleep time (specifically, increase in the amount of REM sleep, which has been shown to decrease the emotional reactivity of amygdala) are generally considered as appropriate forms of emotional regulation. These regulation strategies can be taught, either through cognitive behavioral therapy or other forms of psychotherapy aimed at positive emotion regulation.

Of all these strategies, cognitive reappraisal is generally considered to be the best way to reduce sensitivity in emotionally charged situations [245], and this has been confirmed by studies using evoked potentials. For example, negative images usually lead to a stronger response, or higher amplitude of the P300 potential, than neutral images. This is thought to be due to the fact that the P300 reflects the degree of attention given to the stimulus [246,247], and it is always higher for emotionally charged stimuli. However, if respondents are taught to adjust their own assessment of displayed images or videos by looking at them in a more positive way, they will overcome such stress in a much easier way, as confirmed by the lower amplitude of the P300 potential [248,249].

Research using fMRI can also help identify areas of the brain (“sources”) that are involved in performing this conscious emotional control (top-down control), as well as those areas whose activity is modulated by such control (“goals”) [250]. For example, in a situation when facing a unpleasant medical procedure in a hospital one can try to reduce anxiety by mentally distancing from the situation, e.g., by imagining lying down in a more pleasant setting. Such a strategy will usually reduce the degree of perceived pain, and is mediated by the cerebral cortex as the “source” of control as its activity increases during the exposure to the situation, while activity in subcortical areas (“presumed targets”) decreases [251,252]. In another experimental paradigm, in which men were shown explicitly challenging images and instructed to try to suppress their sexual arousal, the strongest increase in activity was observed in the superior frontal gyrus of the right hemisphere, and a decrease in activity was documented in the hypothalamus and amygdala [253,254]. Likewise, when subjects were instructed to re-evaluate disturbing images, increase in the activity in the frontal lobe, and decrease in the amygdaloid nucleus was detected [255]. In another similar paradigm in which subjects were required to suppress emotionally charged memories, increased activity of the lower and middle frontal gyri of the right hemisphere was noted, together with reduced activity of the hippocampus and amygdaloid nucleus [256]. Although the results of these studies are quite similar, the main problem with these neuroimaging findings is the fact that such studies can only provide answers to questions about whether the activity of a part of the brain correlates with a certain cognitive state, but cannot give a definitive answer as to whether a certain part of the cerebral cortex is really the cause of successful regulation of emotions or just a side effect (consequence) of that regulation. To overcome this limitation, one study used dorsolateral prefrontal cortex stimulation with direct transcranial direct current stimulation [257]. Such stimulation improved the effectiveness of cognitive reappraisal as a strategy for emotional control, which was also confirmed by measuring galvanic skin conductivity. It was therefore concluded that the increase in dorsolateral prefrontal cortex activity has a positive cause-and-effect effect on emotion regulation. Since such cognitive assessment activity requires additional frontal cortex resources, it is not uncommon that it reduces the effectiveness of executive and comportment abilities [258]. Stress also negatively affects frontal lobe activity, which explains why a strategy to change cognitive assessment in real-life stressful circumstances is often ineffective [259,260]. Therefore, it can be concluded that by impairing the cognitive regulation of emotion, stress increases vulnerability to psychiatric disorders and age-related neurodegeneration.

## 8. Conclusions

The phenomenon of animal domestication was first described in detail by Charles Darwin, but both assumptions by which he tried to explain it did not prove to be correct. The neural crest hypothesis, which states that the phenotypic features of the domestication syndrome are due to a reduced number or disruption of neural crest cell migration, is now considered the most likely mechanism, as these cells differentiate at their final destinations and proliferate into different tissues whose activity is reduced by domestication. Comparing the phenotypes of modern and prehistoric man, it is obvious that during their recent evolutionary past, humans also went through a process of self-domestication with a simultaneous prolongation of the period of socialization. This has led to the development of social abilities, skills, and specific functions such as language, as well as neoteny (pedomorphosis, fetalization). While anomalies of neural crest cell development and migration lead to many diseases and syndromes in humans for which pathogenesis is well-known. Understanding self-domestication and its cognitive and emotional underpinnings is important considering that self-domestication may influence Williams-Beuren syndrome and schizophrenia (which have the characteristics of hyperdomestication) and autism spectrum disorders and 7dupASD syndrome (which have the characteristics of hypodomestication) that are still poorly investigated. As central states, individual emotions have likely evolved through the influence of natural selection so that by expanding the repertoire of behaviors, they lead to coordinated cognitive, physiological, and behavioral responses that increase the likelihood of survival and reproduction. Negative emotions (fear, anxiety, pain, sadness, depression, jealousy, grief, anger, guilt, shame) are mainly associated with an increased likelihood of survival, while positive emotions are mostly associated with increased reproductive success and care for offspring (excitement, joy, pleasure, desire, hope, infatuation, pride, optimism, friendship, love). Using game theory models, social neuroscience studies the emotional interactions between personal and group benefits, for example in the context of competition and cooperation at work, politics, and other social environments. Psychotherapy, cognitive-behavioral therapy, and other procedures for people with emotional disorders seek to help with interventions in different steps of emotional experience. The most effective strategies are cognitive reappraisal of one’s own situation, social distancing, and healthy humor.
